# Coupled-cluster treatment of complex open-shell systems: the case of single-molecule magnets†

**DOI:** 10.1039/d4cp01129e

**Published:** 2024-05-27

**Authors:** Maristella Alessio, Garrette Pauley Paran, Cansu Utku, Andreas Grüneis, Thomas-C. Jagau

**Affiliations:** a Department of Chemistry, KU Leuven Celestijnenlaan 200F B-3001 Leuven Belgium maristella.alessio@kuleuven.be; b Institute for Theoretical Physics, TU Wien Wiedner Hauptstraße 8-10/136 1040 Vienna Austria

## Abstract

We investigate the reliability of two cost-effective coupled-cluster methods for computing spin-state energetics and spin-related properties of a set of open-shell transition-metal complexes. Specifically, we employ the second-order approximate coupled-cluster singles and doubles (CC2) method and projection-based embedding that combines equation-of-motion coupled-cluster singles and doubles (EOM-CCSD) with density functional theory (DFT). The performance of CC2 and EOM-CCSD-in-DFT is assessed against EOM-CCSD. The chosen test set includes two hexaaqua transition-metal complexes containing Fe(ii) and Fe(iii), and a large Co(ii)-based single-molecule magnet with a non-aufbau ground state. We find that CC2 describes the excited states more accurately, reproducing EOM-CCSD excitation energies within 0.05 eV. However, EOM-CCSD-in-DFT excels in describing transition orbital angular momenta and spin–orbit couplings. Moreover, for the Co(ii) molecular magnet, using EOM-CCSD-in-DFT eigenstates and spin–orbit couplings, we compute spin-reversal energy barriers, as well as temperature-dependent and field-dependent magnetizations and magnetic susceptibilities that closely match experimental values within spectroscopic accuracy. These results underscore the efficiency of CC2 in computing state energies of multi-configurational, open-shell systems and highlight the utility of the more cost-efficient EOM-CCSD-in-DFT for computing spin–orbit couplings and magnetic properties of complex and large molecular magnets.

## Abstract 2

Coupled-cluster methods effectively describe spin states and magnetic behavior of large and complex molecular magnets.

## Graphical abstract

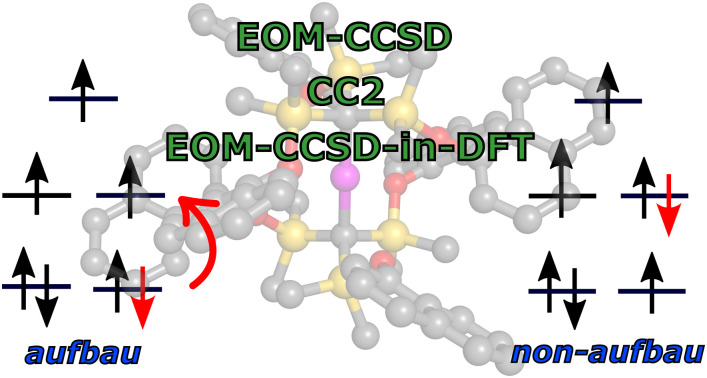

## I. Introduction

The application of quantum chemical methods to investigate electronic states and properties of transition-metal complexes and related materials is a crucial research area, finding applications in catalysis,^1–4^ car-battery design,^5–7^ molecular magnetism^8–10^ and many other fields. In the design of single-molecule magnets (SMMs) with desired behavior, meaning slow magnetic relaxation, the target quantity is the spin-reversal energy barrier.^11^ This barrier influences how effectively a system can be magnetized by an applied magnetic field, as measured by its magnetic susceptibility, and governs the rate of magnetization switching. To maximize this barrier, a rational tuning of the ground state spin *S* and orbital angular momentum *L*, which gives rise to spin–orbit coupling (SOC), is key.^12^ In transition-metal SMMs, the ligand field typically removes molecular-orbital degeneracy, thereby suppressing the orbital angular momentum. However, compounds with a weak ligand field and an odd number of unpaired electrons for each d shell may have unquenched orbital angular momentum, substantial SOC, and high spin-reversal barrier. This phenomenon has been observed in a set of linear SMMs with twofold-coordinated Fe(ii),^13^ Fe(i),^14^ and Co(ii)^15^ metal centers. Among them, the Co(C(SiMe_2_ONaph)_3_)_2_ complex stands out with the highest barrier, reaching 450 cm^−1^.^15^ However, building such linear coordination environment necessitates bulky naphthyl (Naph) groups as ligands. Furthermore, the development of molecular quantum devices involves designing relatively large and complex systems, obtained by deposition of molecular magnets on a surface or their self-assembly.^16^ Alongside the structural complexity of these magnetic systems, the small energy gaps, from tens to hundreds of wavenumbers, call for spectroscopic accuracy rather than chemical accuracy. As the focus in molecular magnetism turns toward transition metals coordinated by increasingly large ligands, often binding with various environments, there is a growing demand for quantum chemical methods that are both efficient and reliable in characterizing these systems.^17,18^

Describing open-shell electronic states is typically more challenging compared to closed-shell electronic states, and standard single-reference methods, such as density functional theory (DFT), are often qualitatively incorrect. Traditionally, multi-reference methods, *e.g.*, complete active space perturbation theory (CASPT2)^19^ and *n*-electron valence-state perturbation theory (NEVPT2),^20^ are employed to approximate the exact multi-configurational wave functions of these systems, extracting magnetic properties through phenomenological spin Hamiltonians.^9,10,21^ For example, NEVPT2 calculations on the Co(C(SiMe_2_ONaph)_3_)_2_ SMM predicted a non-aufbau ground state with *S* = 3/2, *L* = 3, and total angular momentum *J* = *S* + *L* = 9/2, also providing accurate estimates of its magnetic properties.^15^ An alternative approach to treating transition-metal SMMs is offered by the equation-of-motion coupled-cluster (EOM-CC) method,^22–24^ which extends the hierarchy of black-box single-reference coupled-cluster methods to strongly correlated systems. Recently, Alessio and Krylov introduced a computational protocol for describing transition-metal molecular magnets based on the EOM-CC framework.^25^ The approach is implemented in the *ezMagnet* software.^25^ It allows for the computation of spin properties such as spin–orbit splittings, magnetizations, and susceptibilities based on EOM-CC eigenstates. The performance of the computational approach has been tested against experiments and NEVPT2 calculations for a set of Fe(iii), Fe(ii) and Ni(ii) molecular magnets, illustrating its reliability in computing magnetic exchange interactions, energy barriers, and magnetizations and susceptibilities.^18,25,26^ However, the computational cost of EOM-CC methods scales steeply with the system size, rendering their application to large molecular magnets impractical.

In this work, we tackle this challenge by assessing the performance of two more computationally efficient CC methods: the second-order approximate coupled-cluster singles and doubles (CC2)^27^ method, and projection-based embedding,^28^ which combines EOM-CC singles and doubles (EOM-CCSD) with DFT, to treat open-shell transition-metal complexes. The computational efficiency gain of CC2, which scales as *N*^5^ compared to *N*^6^ for EOM-CCSD where *N* is the system size, is achieved through a perturbative analysis of the double amplitude equations. Typically, CC2 is paired with either the resolution-of-the-identity (RI) approximation^29^ or Cholesky-decomposition (CD)^30^ of the electron repulsion integrals, thereby enabling its application to even larger molecules. Although CC2 has been extensively applied in the study of excited states,^23,27,29–35^ its application to multi-configurational wave functions is scarce.

Recently, some of us introduced a spin–flip (SF) variant^36^ of the CC2 method. In SF approaches,^37–39^ multi-configurational lower-spin states are obtained by spin–flipping excitations using a single-determinant high-spin state as reference. The advantage of the SF approach lies in its capability to provide a balanced treatment of all relevant spin states, capturing both dynamical and non-dynamical correlation within a single computational step. Besides the original excitation energy (EE)-CC2 method and its SF-CC2 variant, further CC2 methods are also available. These include the ionization potential^40–42^ (IP) and the electron attachment^42–44^ (EA) variants, where the number of electrons in the target state is decreased or increased by one compared to that of the reference wave function. These EE, SF, IP, and EA variants^36,44^ of the CC2 method are available in the *Q-Chem* software^45^ for restricted (RHF), unrestricted (UHF), and restricted open-shell (ROHF) reference wave functions, providing access to a wide range of electronic structure patterns.

Alternatively, quantum embedding theories^28,46–49^ afford a reduction in computational effort by exploiting the locality of the chemical phenomenon under study. These theories combine different levels of quantum chemical calculations, a more accurate high-level treatment for the active region and a more approximate low-level one for the environment. Among these approaches, projection-based embedding^28,48,50,51^ has emerged as a popular choice for studying both ground and excited states^52^ in isolated molecules as well as periodic systems.^53^ In projection-based embedding, the electron density of the high-level fragment is optimized in the presence of an external embedding potential, built from the electron density of the low-level region. This embedding scheme enforces orthogonality between fragment orbitals *via* a projection technique, removing the need for non-additive kinetic energy potentials. By keeping the electron density of the environment frozen, the approach assumes that the chemical phenomenon of interest is localized in a restricted region, which is often the case for transition-metal complexes whose unpaired electrons are localized on the metal center.^9^ Recently, projection-based embedded EOM-CCSD has been shown to describe very well ionization and valence excitations in small organic molecules microsolvated by water.^54^ Similarly, other embedding schemes have emerged, employing the pair coupled-cluster doubles (pCCD) ansatz^55,56^ as high-level method for addressing excited states in large molecules.^57^ To treat relativistic effects in heavier elements, methods based on four-component Hamiltonians, have also been proposed.^58^ Moreover, projection-based embedding has been extended to describe open-shell electronic states of transition-metal compounds,^59^ but its applications are still somewhat limited. This method is available in the *Q-Chem* software within an unrestricted formalism, which closely follows the original formulation presented in ref. 59. One advantage of this projection-based embedding is its seamless integration into the EOM-CC framework. Computing the respective electronic states entails no further coding or development work, provided that the high-level fragment's orbitals are re-optimized in a self-consistent manner in the presence of the external embedding potential. Consequently, most functionalities inherent to EOM-CC are also readily accessible for embedded EOM-CC. This includes spin-state analysis, as well as the calculation and analysis of the spin properties using reduced quantities, *i.e.*, the one-particle density matrix and corresponding natural orbitals (NO).^60–67^

To evaluate the performance of CC2 and EOM-CCSD-in-DFT, we compute state energies and spin-related properties of a set of transition-metal complexes, exhibiting various electron configurations, specifically, d^5^, d^6^, and d^7^. Our test set comprises: (i) two iron aqua complexes, [Fe(H_2_O)_6_]^3+^ and [Fe(H_2_O)_6_]^2+^, (ii) a single-center cobalt-based SMM, Co(C(SiMe_2_ONaph)_3_)_2_,^15^ and (iii) its simplified model, Co(C(SiH_3_)_3_)_2_. The [Fe(H_2_O)_6_]^2+^ and [Fe(H_2_O)_6_]^3+^ complexes are commonly used as benchmarks for investigating spin-state splittings in transition-metal complexes and oxide materials;^68–70^ however, this is the first study reporting SOCs. On the other hand, the Co(C(SiMe_2_ONaph)_3_)_2_ SMM poses great computational challenges due to its unconventional non-aufbau filling of the d orbitals^15^ and large number of electrons (681). In addition to state energies and SOCs, we derive spin–orbit splittings, magnetizations, and susceptibilities using the protocol implemented in the *ezMagnet* software. The performance of CC2 and EOM-CCSD-in-DFT is assessed through a comparison with results from EOM-CCSD calculations and experiments.^15^ In addition to CC2 and EOM-CCSD-in-DFT, we also consider the combination of the SF approach with time-dependent DFT (SF-TD-DFT),^71,72^ which has proven to be an efficient yet reliable alternative to EOM-CCSD for transition-metal molecular magnets.^18,25,26,73^ This work also marks the first application of EOM-CC methods to non-aufbau electron configurations of 3d transition-metal compounds. Furthermore, it provides guidelines for determining where one variant (EE, SF, EA, or IP) of CC2 or EOM-CCSD-in-DFT is more suitable than another, and serves as inspiration for further implementation efforts aimed at improving the generality and efficiency of these methods.

## II. Theoretical and computational details

### A. Molecular structures

**Fig. 1:**
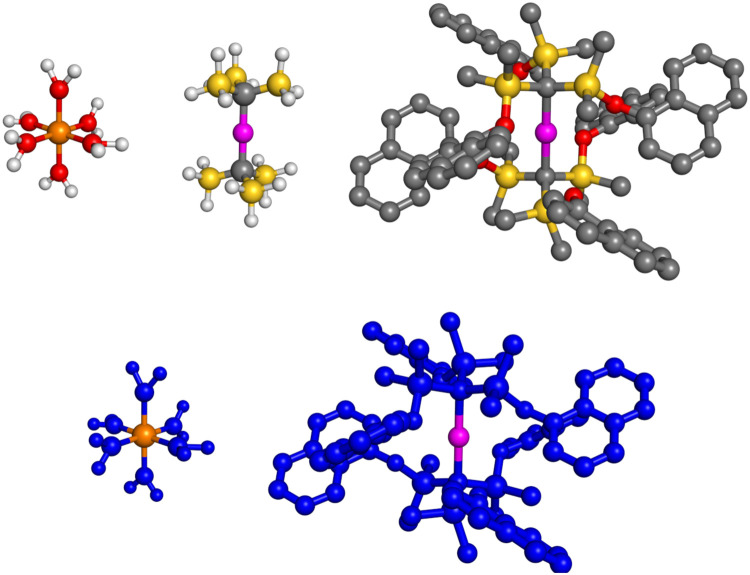
Top: Structures of [Fe(H_2_O)_6_]^2+^ and [Fe(H_2_O)_6_]^3+^, Co(C(SiH_3_)_3_)_2_, and Co(C(SiMe_2_ONaph)_3_)_2_ complexes. For Co(C(SiMe_2_ONaph)_3_)_2_, the hydrogen atoms are omitted. Color code: Co – magenta, Fe – orange, Si – yellow, O – red, C – gray, H – white. Bottom: Partitioning of [Fe(H_2_O)_6_]^2+^, [Fe(H_2_O)_6_]^3+^, and Co(C(SiMe_2_ONaph)_3_)_2_ complexes into high-level fragment (orange and magenta for Fe and Co, respectively) and low-level fragment (blue) for the embedded EOM-CCSD calculations.

The structural models of the systems under study are depicted in Fig. 1. [Fe(H_2_O)_6_]^2+^ and [Fe(H_2_O)_6_]^3+^ are sufficiently small to be treated as a whole by EOM-CCSD. These compounds exhibit *T*_h_ point group symmetry, which is commonly handled through its Abelian subgroup *D*_2h_. For [Fe(H_2_O)_6_]^2+^, the degeneracy of two e_g_ orbitals (a_g_ in *D*_2h_) results in an electronically excited doubly-degenerate ^5^E_g_ state, which corresponds to two degenerate ^5^A_g_ states in *D*_2h_. There exists no subgroup of *T*_h_ where E_g_ can split into two distinct irreducible representations (irreps). Therefore, the two degenerate states belong to the same irrep, rendering them indistinguishable in an EOM-CCSD calculation based on real algebra and the *D*_2h_ point group symmetry. Using complex algebra and the *T*_h_ point group, there would be no problem as the two E_g_ states then had different complex-valued characters. However, here we adopted a slightly distorted structure for [Fe(H_2_O)_6_]^2+^ to overcome these issues, following the strategy outlined in ref. 74. This structure is optimized with *ω*B97X-D/cc-pVDZ, whereas for [Fe(H_2_O)_6_]^3+^, we exploited symmetry and used its *T*_h_ geometry, optimized with *ω*B97X-D/cc-pVDZ.

The calculation of spin states and properties of Co(C(SiMe_2_ONaph)_3_)_2_ was computationally feasible only using EOM-CCSD-in-DFT. The structure of Co(C(SiMe_2_ONaph)_3_)_2_ was taken from ref. 15 (*C*_1_ point group symmetry). We also considered a model system, *i.e.*, Co(C(SiH_3_)_3_)_2_, representing Co(C(SiMe_2_ONaph)_3_)_2_, which is derived from the original complex by replacing methyl and naphthyl groups in the ligands with hydrogen atoms. Co(C(SiH_3_)_3_)_2_ has *D*_3d_ symmetry; however, our calculations were carried out in the C_2h_ point group. The molecular structure of Co(C(SiH_3_)_3_)_2_ was optimized with *ω*B97X-D/cc-pVDZ. By making use of symmetry, both EOM-CCSD and CC2 calculations for Co(C(SiH_3_)_3_)_2_ were feasible. All relevant Cartesian coordinates are given in the ESI.†

### B. Electronic structures

**Fig. 2:**
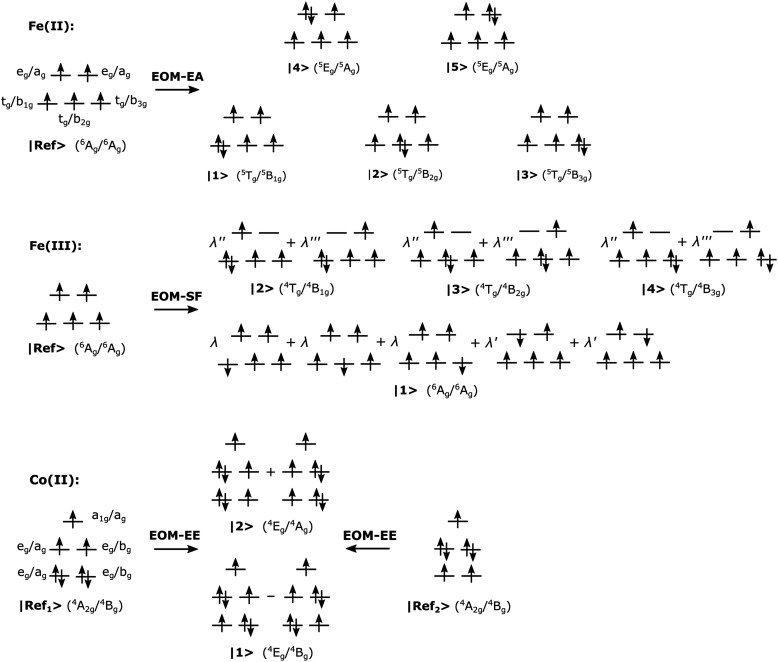
Simplified electron configurations of the reference and target states for [Fe(H_2_O)_6_]^2+^ (top), [Fe(H_2_O)_6_]^3+^ (middle), and Co(C(SiH_3_)_3_)_2_ and Co(C(SiMe_2_ONaph)_3_)_2_ (bottom). For [Fe(H_2_O)_6_]^2+^ and [Fe(H_2_O)_6_]^3+^, *T*_h_ (and *D*_2h_) irreps are used. For Co(C(SiH_3_)_3_)_2_ and Co(C(SiMe_2_ONaph)_3_)_2_, *D*_3d_ (and *C*_2h_) irreps are used. For [Fe(H_2_O)_6_]^3+^, *λ* = 0.40, *λ*′ = 0.34, and *λ*′′ and *λ*′′′ are arbitrarily assigned.

Fig. 2 summarizes the electronic structure of the Fe and Co complexes and illustrates which variants of the EOM-CCSD, CC2, and EOM-CCSD-in-DFT methods are used to access the target states. Note, however, that the electronic structure of the iron complexes is simplified in Fig. 2 as there are actually two e_g_ shells that result from bonding and antibonding interaction of the metal d-orbitals with the ligand orbitals. Additionally, for the Co(ii)-SMM, Table 1 presents the electron configurations of its states. To facilitate the discussion, a numerical labeling is used for all states. However, for the Fe(ii) and Fe(iii) complexes, Fig. 2 provides term symbols for *T*_h_ symmetry and its subgroup *D*_2h_ as well. For the linear and twofold-coordinated Co(ii) molecular magnet, we also included term symbols for the *D*_3d_ and *C*_2h_ point groups. Moreover, in ref. 15, NEVPT2 states of the Co(ii)-SMM were classified using the *C*_∞v_ point group notation. Therefore, to facilitate comparison with these NEVPT2 results, term symbols for *C*_∞v_ symmetry are also included in Table 1.

**Table:** Electron configurations of the reference (|Ref_1_〉 and |Ref_2_〉) and target states, and computed transition angular momenta for Co(C(SiH_3_)_3_)_2_ (EOM-EE-CCSD/cc-pVTZ). States |1〉, |2〉, |3〉, and |4〉 are obtained by single-electron excitation of a β-electron from |Ref_1_〉. States |1〉, |2〉, |3′〉, |4′〉, |5′〉, and |6′〉 are obtained by single-electron excitation of a β-electron from |Ref_2_〉. Term symbols using *C*_∞v_ symmetry are also reported, following the notation used in ref. 15

State	Term (*C*_∞*v*_)	Configuration	〈*L*_*z*_〉
|Ref_1_〉	^4^Σ^−^	(d_*xy*_, d_*x*^2^−*y*^2^_)^4^ (d_*xz*_, d_*yz*_)^2^ (d_*z*^2^_)^1^	
|1〉, |2〉	^4^Φ	(d_*xy*_, d_*x*^2^−*y*^2^_)^3^ (d_*xz*_, d_*yz*_)^3^ (d_*z*^2^_)^1^	〈1|*L*_*z*_|2〉 = 2.98*i*
|3〉, |4〉	^4^Δ	(d_*xy*_, d_*x*^2^−*y*^2^_)^3^ (d_*xz*_, d_*yz*_)^2^ (d_*z*^2^_)^2^	〈3|*L*_*z*_|4〉 = 1.99*i*

|Ref_2_〉	^4^Σ^−^	(d_*xy*_, d_*x*^2^−*y*^2^_)^2^ (d_*xz*_, d_*yz*_)^4^ (d_*z*^2^_)^1^	
|1〉, |2〉	^4^Φ	(d_*xy*_, d_*x*^2^−*y*^2^_)^3^ (d_*xz*_, d_*yz*_)^3^ (d_*z*^2^_)^1^	〈1|*L*_*z*_|2〉 = 2.98*i*
|3′〉, |4′〉	^4^Π	(d_*xy*_, d_*x*^2^−*y*^2^_)^3^ (d_*xz*_, d_*yz*_)^3^ (d_*z*^2^_)^1^	〈3′|*L*_*z*_|4′〉 = 1.00*i*
|5′〉, |6′〉	^4^Π	(d_*xy*_, d_*x*^2^−*y*^2^_)^2^ (d_*xz*_, d_*yz*_)^3^ (d_*z*^2^_)^2^	〈5′|*L*_*z*_|6′〉 = 0.99*i*

For [Fe(H_2_O)_6_]^2+^ and [Fe(H_2_O)_6_]^3+^, we computed the excitation energy between the ground state and the first electronically excited state at the equilibrium structure of the [Fe(H_2_O)_6_]^3+^ high-spin sextet ground state. The ground state of [Fe(H_2_O)_6_]^2+^ is a triply-degenerate quintet state (the states |1〉, |2〉, and |3〉 in Fig. 2), with a low-lying doubly-degenerate quintet excited state (states |4〉 and |5〉). [Fe(H_2_O)_6_]^3+^ has a spatially non-degenerate ground state (the state |1〉) and a triply-degenerate quartet excited state (states |2〉, |3〉, and |4〉). To treat the quintet d^6^ target states of [Fe(H_2_O)_6_]^2+^, we used the EA variant of the CC methods with a d^5^ high-spin sextet reference. On the contrary, for the sextet and quartet d^5^ states of [Fe(H_2_O)_6_]^3+^, we used the SF approach with the same d^5^ high-spin sextet reference. For the Co(ii)-SMM, we computed four doubly-degenerate quartet states: states |1〉 and |2〉, states |3〉 and |4〉, states |3′〉 and |4′〉, and states |5′〉 and |6′〉. These states are treated using the EE approach, in which a β-electron is excited from the fully occupied and degenerate d-orbital shells, either (d_*xy*_, d_*x*^2^−*y*^2^_)^4^ or (d_*xz*_, d_*yz*_)^4^, of the reference states, |Ref_1_〉 and |Ref_2_〉 in Fig. 2, respectively.

### C. Details of the electronic-structure calculations

Fig. 1 shows the partitioning scheme adopted in all embedded EOM-CCSD calculations. The [Fe(H_2_O)_6_]^2+^ and [Fe(H_2_O)_6_]^3+^ complexes have 84 and 83 electrons, respectively. Within our partitioning, the electrons associated with the metal center, 24 for [Fe(H_2_O)_6_]^2+^ and 23 for [Fe(H_2_O)_6_]^3+^, constitute the high-level EOM-CCSD fragment (orange in Fig. 1), while the remaining 60 electrons of the water molecules form the low-level DFT fragment (blue in Fig. 1). For Co(C(SiMe_2_ONaph)_3_)_2_, this partitioning is even more accentuated with only 25 electrons ascribed to the high-level EOM-CCSD fragment (magenta in Fig. 1) and 656 to the low-level DFT fragment (blue in Fig. 1). Fig. S1 (ESI†) illustrates the spin difference density computed using the *α* and *β* charge densities. The excess spin density is localized on the metal center, supporting the partitioning we chose. A similar partitioning scheme was previously employed in a CCSD-in-DFT study exploring the dissociation curve of hexaaquairon(ii) cations.^59^ Additionally, to be able to perform embedded EOM-CCSD calculations on the large Co(ii)-SMM, it was critical to truncate the virtual orbital space. This was done using concentric localization.^75^ On the other hand, for the smaller systems, [Fe(H_2_O)_6_]^2+^, [Fe(H_2_O)_6_]^3+^, and Co(C(SiH_3_)_3_)_2_, EOM-CCSD-in-DFT calculations were also feasible without truncation of the virtual space.

Moreover, in the case of [Fe(H_2_O)_6_]^2+^ and [Fe(H_2_O)_6_]^3+^, we investigated the dependence on the density functional in EOM-CCSD-in-DFT calculations, as well as for the SF-TD-DFT calculations, using the hybrid functionals PBE0,^76^ B3LYP,^77^ and B5050LYP,^71^ along with CAM-B3LYP,^78^*ω*B97X-D,^79^ and LRC-*ω*PBEh^80^ as range-separated hybrid functionals. Following previous SF studies of SMMs,^26,73^ we employed the non-collinear formulation of SF-TD-DFT^72,81^ to calculate spin states and spin-related properties. For Co(C(SiH_3_)_3_)_2_ and Co(C(SiMe_2_ONaph)_3_)_2_, we used LRC-*ω*PBEh as low-level method. However, replacing LRC-*ω*PBEh by CAM-B3LYP for Co(C(SiH_3_)_3_)_2_ does not affect the spin-state ordering (see Table S8, ESI†). The differences observed between EOM-CCSD-in-LRC-*ω*PBEh and EOM-CCSD-in-CAM-B3LYP results are less than 500 cm^−1^ for state energies and within 50 cm^−1^ for the SOCs.

In our CC2 implementation, all CC2 variants^36,44^ can be combined with either the resolution-of-the-identity (RI) approximation^29^ or Cholesky decomposition (CD)^30^ of the electron repulsion integrals. For the Fe(ii) and Fe(iii) complexes, we carried out RI-EA-CC2 and RI-SF-CC2 calculations, respectively, while we employed CD-EE-CC2 for the Co(ii)-SMM. Table S3 (ESI†) shows that using either the RI or CD approximation has a negligible impact on the excitation energies of the Fe(ii) and Fe(iii) compounds. In addition, RI-EA-CC2 calculations for Fe(ii) were performed using the spin-component-scaled (SCS) analogue.^82^ This spin-scaled version of CC2 has exhibited superior performance to standard CC2 in computing vertical ionization potentials and electron affinities when benchmarked against EOM-CCSD and CCSD(T).^42,44^ Our present work represents the first application of spin-scaled CC2 to open-shell systems.

In all calculations, the cc-pVTZ basis set^83–85^ was employed unless for the SOC calculations of the actual Co(ii)-SMM where we used the 6-31G* basis set. The corresponding auxiliary basis set^86^ was used for the RI-CC2 calculations, while we used a threshold of 10^−3^ for the Cholesky decomposition. The core electrons were frozen in all calculations. Open-shell reference states were computed using unrestricted Hartree–Fock (UHF). However, the level of spin contamination in the reference and target states is minimal: the corresponding 〈*S*^2^〉 values are between 6.00 and 6.19 for the quintet states of Fe(ii), between 8.75 and 8.20 for the sextet states of Fe(iii), between 3.77 and 3.82 for the quartet states of Fe(iii), and between 3.75 and 3.92 for the quartet states of Co(ii) (Tables S1, S2 and S4 in the ESI†). All electronic-structure and spin–property calculations were carried out using the *Q-Chem* program package, version 6.0.^45^

### D Wave function analysis and magnetic properties

To characterize the nature of the electronic states of the Fe and Co complexes, we employed density-based analysis using natural orbitals (NOs) and natural-transition orbitals (NTOs), *i.e.*, eigenvectors of the one-particle state- and transition-density matrices, respectively, along with their corresponding eigenvalues.^60–66^ For the state analysis, these eigenvalues can be interpreted as the occupation numbers of each NO. On the other hand, when analyzing NTOs, the eigenvalues of the transition density matrix are the weights of each NTO hole–particle pair describing the electronic excitation between the states. This type of analysis has been extensively applied in a set of transition-metal complexes including Fe(iii), Fe(ii), and Ni(ii) with varying numbers of unpaired electrons and diverse electronic structure patterns (d^5^, d^6^, d^8^), using methods such as EOM-CCSD and SF-TD-DFT.^18,25,26,67^ Building upon these previous works, we extended our density-based analysis to include EOM-CCSD-in-DFT and the non-aufbau ground state of the Co(ii)-SMM.

In addition to computing state energies, we focused on orbital angular momenta and spin–orbit coupling constants (SOCCs), which are given by the following expression:1
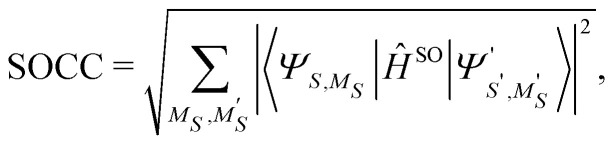
where the sum runs over all multiplet components with spin projection *M*_*S*_ and 
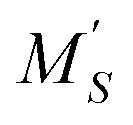
 of the spin–orbit coupled states *Ψ* and *Ψ*′. The SOCs entering eqn (1) are expressed as matrix elements of the Breit-Pauli spin–orbit Hamiltonian^87,88^ evaluated for non-relativistic EOM-CC wave functions, as implemented in the *Q-Chem* software by Krylov and co-workers.^74,89,90^ This procedure has been demonstrated to accurately treat SOC in 3d transition-metal systems.^18,25,26,74^ Within this implementation, transition properties are computed as contraction of the corresponding integrals with reduced transition density matrices, therefore their calculation is general and can be interfaced with any method providing density matrices, *e.g.*, EOM-CCSD, SF-TD-DFT, and EOM-CCSD-in-DFT.^74,89,90^ To evaluate the reliability of EOM-CCSD-in-DFT for spin properties, we computed SOCCs for [Fe(H_2_O)_6_]^2+^ and [Fe(H_2_O)_6_]^3+^, for which full EOM-CCSD calculations on the whole systems are viable as references. In the case of [Fe(H_2_O)_6_]^2+^, we computed the SOCC between the three degenerate states |1〉, |2〉, and |3〉. For [Fe(H_2_O)_6_]^3+^, we computed the SOCC between the ground state (state |1〉) and the triply-degenerate excited state (states |2〉, |3〉, and |4〉). In addition, we evaluated the SOCC for the doubly-degenerate ground state of Co(C(SiH_3_)_3_)_2_ (states |1〉 and |2〉) using both EOM-CCSD and EOM-CCSD-in-DFT. On the contrary, in the case of Co(C(SiMe_2_ONaph)_3_)_2_, SOCC calculations were only possible using EOM-CCSD-in-DFT with a smaller basis set, 6-31G*. However, energy calculations for Co(C(SiMe_2_ONaph)_3_)_2_ using EOM-CCSD-in-DFT were also performed with the cc-pVTZ basis set (see Table S10, ESI†). Using the larger cc-pVTZ basis set, the ordering of the states remains unchanged, with relative changes ranging from 200 cm^−1^ for the low-lying excited states to up to 1000 cm^−1^ for the highest excited states |5′〉 and |6′〉.

**Fig. 3:**
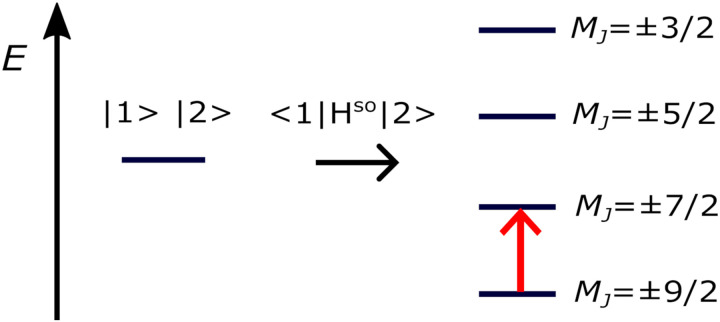
Spin–orbit splitting of the *J* = 9/2 ground state (states |1〉 and |2〉) of Co(C(SiH_3_)_3_)_2_ and Co(C(SiMe_2_ONaph)_3_)_2_. The energy barrier for spin-inversion is shown in red.

Furthermore, using the *ezMagnet* software,^25^ we computed magnetic properties of the Co(ii)-SMM, *i.e.*, the spin-reversal energy barrier, magnetization, and susceptibility, allowing for the direct comparison with the experiment. Magnetic properties arise from spin–orbit and Zeeman interactions. To account for these effects, we employed a two-step state-interaction scheme:^91–93^ first we computed EOM-CCSD or EOM-CCSD-in-DFT states and then used these states to evaluate matrix elements of the spin–orbit (*Ĥ*^SO^) and Zeeman (*Ĥ*^Z^) operators. Magnetic sublevels are then obtained by diagonalizing the perturbed Hamiltonian, *i.e.*, *Ĥ* = *Ĥ*^0^ + *Ĥ*^SO^ + *Ĥ*^Z^, where *Ĥ*^0^ is the Born–Oppenheimer Hamiltonian in the basis of EOM-CC states. Subsequently, by applying Boltzmann statistics, the magnetization and susceptibility are obtained from the resulting partition function. Following this protocol, we computed the spin–orbit levels of the Co(ii)-SMM by accounting for the SOC between states |1〉 and |2〉. Due to SOC, the doubly-degenerate ground state (states |1〉 and |2〉) of the Co(ii)-SMM splits into four Kramers doublets,^15^ as shown in Fig. 3. This, in turn, allowed us to quantify the spin-reversal barrier as the energy separation between the ground *M*_*J*_ = ±9/2 state and the first excited *M*_*J*_ = ±7/2 state, where *M*_*J*_ is the projection of *J* along the magnetic axis. This strategy has been already employed in a number of studies^15,94^ assuming that magnetic relaxation occurs by a combination of the Orbach mechanism^95,96^ and quantum tunnelling from the first excited state.

## III. Results and discussion

### A. Fe(ii) and Fe(iii) complexes

**Fig. 4:**
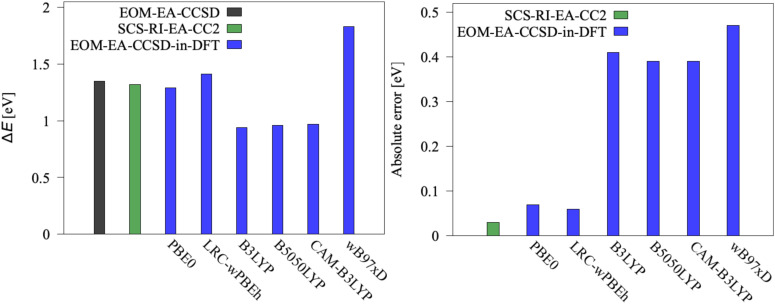
Left: Excitation energy Δ*E* for [Fe(H_2_O)_6_]^2+^ (|1〉, |2〉, |3〉 → |4〉, |5〉) computed using EOM-EA-CCSD, SCS-RI-EA-CC2, and EOM-EA-CCSD-in-DFT with cc-pVTZ basis set. Right: Absolute errors of EOM-EA-CCSD-in-DFT and SCS-RI-EA-CC2 with respect to EOM-EA-CCSD. EOM-EA-CCSD-in-DFT energies are obtained without truncation of the virtual orbital space.

**Fig. 5:**
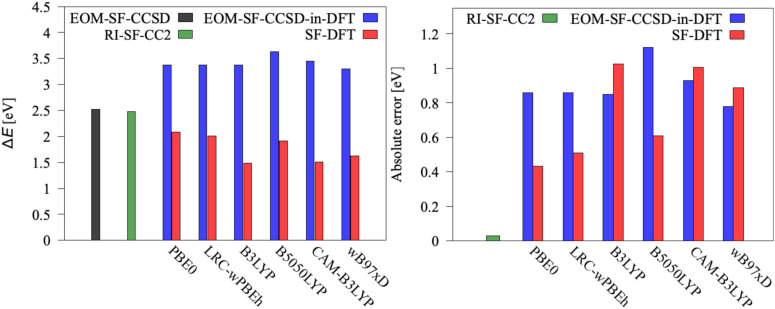
Left: Excitation energy Δ*E* for [Fe(H_2_O)_6_]^3+^ (|1〉 → |2〉, |3〉, |4〉) computed using EOM-SF-CCSD, RI-SF-CC2, EOM-SF-CCSD-in-DFT, and SF-TD-DFT with cc-pVTZ basis set. Right: Absolute errors of EOM-SF-CCSD-in-DFT, RI-SF-CC2, and SF-TD-DFT with respect to EOM-SF-CCSD. EOM-SF-CCSD-in-DFT energies are obtained without truncation of the virtual orbital space.

Fig. 4 and 5 depict excitation energies (left panels) of [Fe(H_2_O)_6_]^2+^ and [Fe(H_2_O)_6_]^3+^, respectively, computed using EOM-CCSD, CC2, and EOM-CCSD-in-DFT. The right panels show absolute errors of CC2 and EOM-CCSD-in-DFT as compared to EOM-CCSD. For [Fe(H_2_O)_6_]^2+^, the SCS-RI-EA-CC2 excitation energy closely agrees with the EOM-EA-CCSD value, with an error of less than 0.05 eV. Furthermore, EOM-EA-CCSD-in-DFT performs relatively well, with errors within 0.1 eV, only when the PBE-based functionals, PBE0 and LRC-*ω*PBEh, are employed as low-level methods. On the contrary, the absolute error increases to 0.4 eV for B3LYP, B5050LYP, and CAM-B3LYP, and to 0.5 eV for *ω*B97X-D. For [Fe(H_2_O)_6_]^3+^, RI-SF-CC2 outperforms again EOM-SF-CCSD-in-DFT in computing excitation energies. Both EOM-SF-CCSD-in-DFT and SF-TD-DFT, regardless of the selected density functional, exhibit poor performance compared to EOM-SF-CCSD, with errors as large as 1 eV. Interestingly, while EOM-SF-CCSD-in-DFT tends to overestimate the excitation energy, SF-TD-DFT underestimates it, both by a similar magnitude, on average +0.9 eV for EOM-SF-CCSD-in-DFT and −0.7 eV for SF-TD-DFT. In all SF calculations, only the *M*_*S*_ = 3/2 state (state |1〉) is used to compute excitation energies and SOCCs, with the high-spin state (*M*_*S*_ = 5/2) serving solely as reference.^18,37,39,97^ However, while the energy difference between the reference state and state |1〉 of [Fe(H_2_O)_6_]^3+^ is 0.05 eV for EOM-CCSD, this difference further deviates from zero for more approximate methods, ranging from 0.1 eV for SF-CC2 to 0.2 eV for SF-TD-DFT, and up to 1 eV for EOM-CCSD-in-DFT. As this difference vanishes in the full CC limit, these numbers illustrate the decreasing quality of the SF target states.

**Fig. 6:**
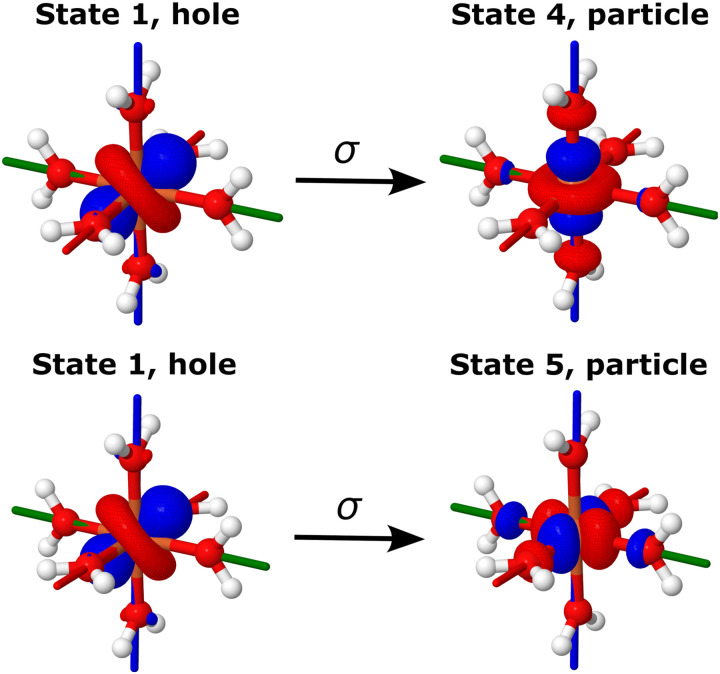
Hole and particle NTOs of the transition density matrix between states |1〉 and |4〉 (top) and |1〉 and |5〉 (bottom) of [Fe(H_2_O)_6_]^2+^ computed with EOM-EA-CCSD-in-LRC-*ω*PBEh/cc-pVTZ. The singular values are *σ* = 0.97 from state |1〉 to state |4〉 and *σ* = 0.97 from state |1〉 to state |5〉. Red, green, and blue axes indicate *x*, *y*, and *z* axes. An isovalue of 0.05 was used.

For correlated many-body theories, NOs and NTOs provide valuable insights into the nature of the wave functions and electron excitations, facilitating the interpretation of the computed properties based on the orbitals involved. In the case of [Fe(H_2_O)_6_]^2+^, transitions between the spin states under study involve a single NTO pair consisting of d-like orbitals, indicating the single-determinantal character of the corresponding electronic states. Fig. 6 illustrates the EOM-CCSD-in-DFT NTOs describing the transitions between the triply-degenerate ground state (|1〉, |2〉, and |3〉) and the doubly-degenerate excited state (|4〉 and |5〉) of [Fe(H_2_O)_6_]^2+^. Specifically, transitions between states |1〉 and |4〉 and between states |1〉 and |5〉 correspond to transitions from d_*yz*_ to d_*z*^2^_ and from d_*yz*_ to d_*x*^2^−*y*^2^_, respectively. Additional NTO pairs for the remaining electronic transitions in [Fe(H_2_O)_6_]^2+^ can be found in Fig. S2 (ESI†). However, for the excitation between the ground state (|1〉) and the excited state (|2〉, |3〉 and |4〉) of [Fe(H_2_O)_6_]^3+^, the squared norm of the one-particle transition density matrix, which is considered a measure of one-electron excitation character,^62,98,99^ is significantly smaller than one (about 0.4), indicative of a substantial two-electron character. As a result, for [Fe(H_2_O)_6_]^3+^, our analysis of states and excitations in terms of NOs and NTOs becomes less meaningful^100^ and is not reported herein.

**Fig. 7:**
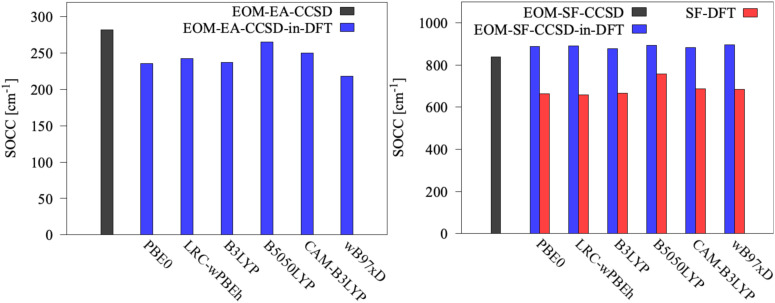
Spin–orbit coupling constants (SOCCs) of [Fe(H_2_O)_6_]^2+^ (left) and [Fe(H_2_O)_6_]^3+^ (right) computed using EOM-CCSD and EOM-CCSD-in-DFT with the cc-pVTZ basis set. For [Fe(H_2_O)_6_]^2+^, the SOCC is computed between states |1〉 and |2〉, |1〉 and |3〉, and |2〉 and |3〉. For [Fe(H_2_O)_6_]^3+^, the SOCC is computed between state |1〉 and the triply-degenerate excited state (*i.e.*, states |2〉, |3〉, and |4〉). EOM-CCSD-in-DFT SOCCs are obtained without truncation of the virtual orbital space.

We also calculate SOCCs using EOM-CCSD, EOM-CCSD-in-DFT, and SF-TD-DFT eigenstates, see eqn (1). Fig. 7 illustrates the results. For [Fe(H_2_O)_6_]^3+^, given the two-electron character of the transition between the state |1〉 and states |2〉, |3〉 and |4〉, and also considering the mean-field treatment of the SOCs in terms of one-particle transition density matrices only,^74^ the absolute magnitude of the corresponding SOCCs should be considered with caution. However, in this case, the spin-flipping excitation between the reference wave function and states |2〉, |3〉 and |4〉 is a one-electron process with SOCC = 817 cm^−1^, which deviates only by 20 cm^−1^ from the SOCC between the state |1〉 and states |2〉, |3〉, and |4〉.

In the case of SOCCs, EOM-CCSD-in-DFT is less sensitive to the choice of the low-level DFT method compared to what we observed for energies. EOM-CCSD-in-DFT underestimates the SOCC of [Fe(H_2_O)_6_]^2+^, while it overestimates the SOCC of [Fe(H_2_O)_6_]^3+^, both by a similar magnitude, on average by about 50 cm^−1^ compared to EOM-CCSD. For [Fe(H_2_O)_6_]^3+^, SF-TD-DFT performs relatively poorly with errors three times as large as those of EOM-SF-CCSD-in-DFT. Similar to what we reported for energies, SOCCs computed with EOM-CCSD-in-DFT and SF-TD-DFT follow opposite trends: EOM-CCSD-in-DFT overestimates the SOCC, whereas SF-TD-DFT underestimates it when compared to EOM-SF-CCSD.

For both [Fe(H_2_O)_6_]^2+^ and [Fe(H_2_O)_6_]^3+^, we also explore the effect of truncating the virtual orbital space on the performance of EOM-CCSD-in-DFT. Table 2 presents the results for EOM-CCSD-in-LRC-*ω*PBEh. We observe that the excitation energies of [Fe(H_2_O)_6_]^2+^, computed with EOM-EA-CCSD-in-DFT, where an additional electron is attached to one of the virtual orbitals, are more sensitive to truncation of the virtual orbital space compared to those of [Fe(H_2_O)_6_]^3+^ computed using SF excitation operators. For [Fe(H_2_O)_6_]^2+^, concentric localization leads to a difference of 0.16 eV in the excitation energy. In contrast, for [Fe(H_2_O)_6_]^3+^, the difference between truncated and non-truncated excitation energies is 2 meV only. Furthermore, the impact of truncating the virtual orbital space on the SOCCs is much smaller compared to what we observed for the energies, with only a 10 cm^−1^ difference for [Fe(H_2_O)_6_]^2+^.

Excitation energies Δ*E* (eV) and spin–orbit coupling constants (cm^−1^) of [Fe(H_2_O)_6_]^2+^ and [Fe(H_2_O)_6_]^3+^ computed using EOM-CCSD, CC2, and EOM-CCSD-in-LRC-*ω*PBEh with the cc-pVTZ basis set. The SOCC of [Fe(H_2_O)_6_]^2+^ is computed between states |1〉 and |2〉, |1〉 and |3〉, and |2〉 and |3〉. The SOCC of [Fe(H_2_O)_6_]^3+^ is computed between state |1〉 and the triply-degenerate excited state (*i.e.*, states |2〉, |3〉, and |4〉). EOM-CCSD-in-LRC-*ω*PBEh energies are obtained with and without truncation of the virtual orbital space

**Table:** 

	[Fe(H_2_O)_6_]^2+^			
	EOM-EA-CCSD	SCS-RI-EA-CC2	EOM-EA-CCSD-in-LRC-*ω*PBEh	
			Truncated	Full
Δ*E*	1.35	1.32	1.57	1.41
SOCC	282		252	242

**Table:** 

	[Fe(H_2_O)_6_]^3+^			
	EOM-SF-CCSD	RI-SF-CC2	EOM-SF-CCSD-in-LRC-*ω*PBEh	
			Truncated	Full
Δ*E*	2.52	2.48	3.37	3.38
SOCC	839		890	890

Experimental excitation energies are available for [Fe(H_2_O)_6_]^2+^ and [Fe(H_2_O)_6_]^3+^ in aqueous solution: these are 1.14^69,101^ and 1.56^102^ eV, respectively. The value of 1.14 eV was obtained by averaging over the energies of the two observed optical transitions at 0.99 and 1.29 eV that result from a Jahn–Teller splitting.^69,101^ These experimental data have been frequently used as a reference for evaluating the performance of explicit or implicit solvent models.^68–70^ From these experimental values, our EOM-CCSD excitation energies deviate by 0.21 eV for [Fe(H_2_O)_6_]^2+^ and 0.96 eV for [Fe(H_2_O)_6_]^3+^. However, solvation effects, which are not considered in the present work, are expected to contribute up to 0.5 eV to the excitation energies.^68^ Furthermore, Reimann and Kaupp^70^ have recently shown that the observed lowest-energy excitation of [Fe(H_2_O)_6_]^3+^ at 1.56 eV must be attributed to the deprotonated [Fe(H_2_O)_5_ OH]^2+^, rather than to the iron(iii) hexaaqua complex in solution. Also, CASPT2 excitation energies^69^ of 1.02 and 2.39 eV were recently reported for isolated [Fe(H_2_O)_6_]^2+^ and [Fe(H_2_O)_6_]^3+^, which deviate from our EOM-CCSD results by 0.13 eV and 0.33 eV, respectively.

Our findings indicate that CC2 yields better excitation energies than EOM-CCSD-in-DFT. On the other hand, EOM-CCSD-in-DFT, independent of the chosen density functional, yields relatively accurate SOCCs. For the energies of [Fe(H_2_O)_6_]^2+^, EOM-EA-CCSD-in-PBE0 and EOM-EA-CCSD-in-LRC-*ω*PBEh afford the best agreement with EOM-EA-CCSD, while for the spin–flip methods both EOM-SF-CCSD-in-DFT and SF-TD-DFT show poor performance and no significant dependence on the density functional. Based on these results, we employ the long-range corrected hybrid functional LRC-*ω*PBEh as low-level method to describe a single-center Co(ii)-SMM with EOM-CCSD-in-DFT. Recently, EOM-EE-CCSD-in-DFT calculations of excited states of small organic molecules microsolvated by water also showed that long-range corrected hybrid functionals, specifically CAM-B3LYP, consistently yield smaller errors compared to GGA or hybrid-GGA functionals,^54^ further supporting our chosen computational settings for the cobalt molecular magnet.

### B. Co(ii) single-molecule magnet

**Fig. 8:**
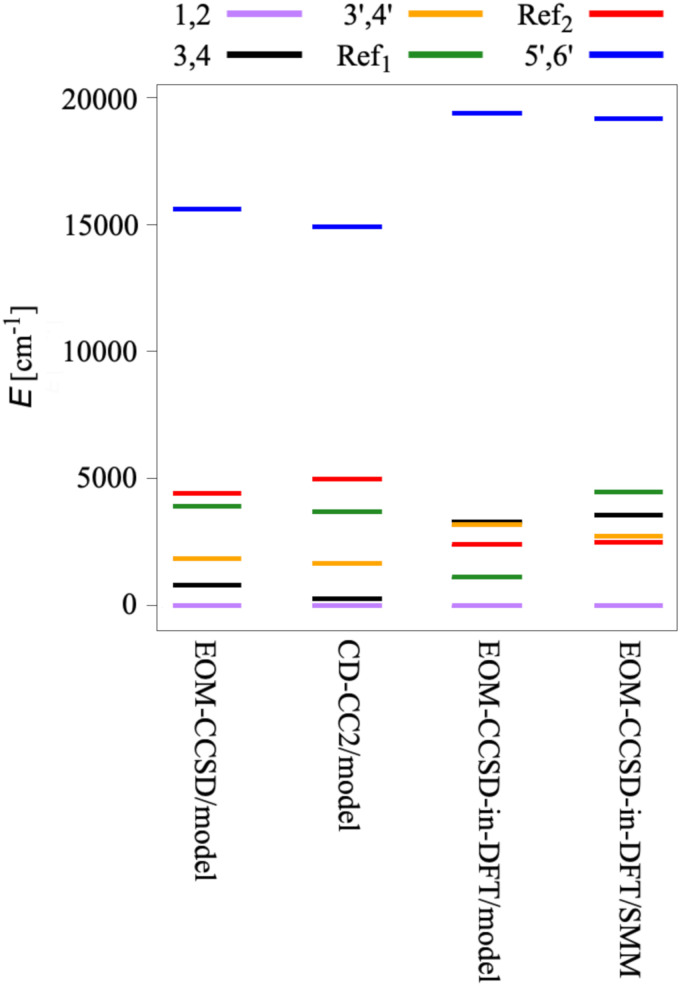
Electronic states of the model system Co(C(SiH_3_)_3_)_2_ and the actual SMM Co(C(SiMe_2_ONaph)_3_)_2_ computed using EOM-EE-CCSD, CD-EE-CC2, and EOM-EE-CCSD-in-LRC-*ω*PBEh. The cc-pVTZ and 6-31G* basis sets were used for Co(C(SiH_3_)_3_)_2_ and Co(C(SiMe_2_ONaph)_3_)_2_, respectively.

**Fig. 9:**
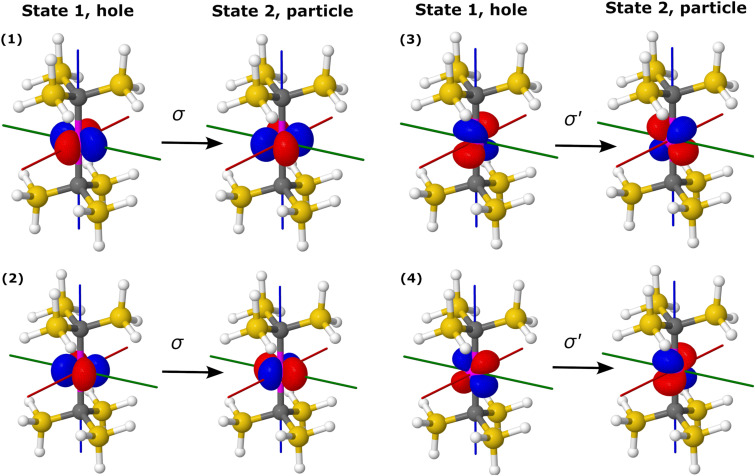
Hole and particle NTOs for SOC between states |1〉 and |2〉 of Co(C(SiH_3_)_3_)_2_ computed with EOM-EE-CCSD-in-LRC-*ω*PBEh/cc-pVTZ. The singular values are *σ* = 0.46 and *σ*′ = 0.45. Red, green, and blue axes indicate *x*, *y*, and *z* axes. An isovalue of 0.05 was used.

For the Co(C(SiMe_2_ONaph)_3_)_2_ molecular magnet, NEVPT2 calculations^15^ revealed that electron configurations following the aufbau principle, such as (d_*xy*_, d_*x*^2^−*y*^2^_)^4^ (d_*xz*_, d_*yz*_)^2^ (d_*z*^2^_)^1^, are less favourable than non-aufbau configurations, *i.e.*, (d_*xy*_, d_*x*^2^−*y*^2^_)^3^ (d_*xz*_, d_*yz*_)^3^ (d_*z*^2^_)^1^, the latter minimizing electron–electron repulsion. The interplay between a weak ligand field and inter-electronic repulsion results in a non-aufbau ground state of maximum orbital angular momentum (*L* = 3) and large spin-reversal energy barrier. Table 3 and Fig. 8 report the energies of the six spin states under study for Co(C(SiH_3_)_3_)_2_ and Co(C(SiMe_2_ONaph)_3_)_2_. All computational methods employed, *i.e.*, EOM-CCSD, CC2, and EOM-CCSD-in-DFT, consistently yield a doubly-degenerate ground state (|1〉 and |2〉) characterized by four main non-aufbau configurations as shown in Fig. 2, which is in agreement with NEVPT2 findings.^15^ According to our NO analysis, the two *β* electrons residing in the d orbitals occupy exclusively the four d_*xy*_, d_*x*^2^−*y*^2^_, d_*xz*_, and d_*yz*_ NOs, each with an occupation number of 0.5 (see Table S5, ESI†). Four NTO pairs contribute to the SOC between states |1〉 and |2〉. These four leading contributions involve a change in orbital orientation from d_*xy*_ to d_*x*^2^−*y*^2^_, as well as from d_*xz*_ to d_*yz*_, as illustrated in Fig. 9 for Co(C(SiH_3_)_3_)_2_ and Fig. S4 (ESI†) for Co(C(SiMe_2_ONaph)_3_)_2_.

**Table:** Relative energies of electronic states (cm^−1^), spin–orbit coupling constants (cm^−1^), and spin-inversion energy barriers *U* (cm^−1^) of Co(C(SiH_3_)_3_)_2_ and Co(C(SiMe_2_ONaph)_3_)_2_ computed using EOM-EE-CCSD, CD-EE-CC2, and EOM-EE-CCSD-in-DFT. For Co(C(SiH_3_)_3_)_2_, EOM-EE-CCSD-in-DFT energies are obtained with and without truncation of the virtual space, for Co(C(SiMe_2_ONaph)_3_)_2_, only with the truncated virtual space

	Co(C(SiH_3_)_3_)_2_				Co(C(SiMe_2_ONaph)_3_)_2_
	EOM-CCSD^a^	CD-CC2^a^	EOM-CCSD-in-DFT^a^^b^		EOM-CCSD-in-DFT^b^^c^
			Truncated	Full	Truncated
|Ref_1_〉	3903	3690	1009	1128	4489
|1〉	0	0	0	0	0
|2〉	11	4	46	39	20
|3〉	803	258	3225	3287	3556
|4〉	803	261	3226	3288	3556

|Ref_2_〉	4427	4964	2400	2412	2492
|1〉	0	0	0	0	0
|2〉	10	1	22	22	45
|3′〉	1855	1645	3187	3184	2721
|4′〉	1855	1657	3187	3184	2722
|5′〉	15 609	14 911	19 406	19 384	19 180
|6′〉	15 609	14 914	19 407	19 384	19 184

〈1|*L*_*z*_|2〉	2.98*i*		3.02*i*	3.01*i*	2.99*i*
SOCC^d^	1126		1114	1107	1059
*U*	504		497	495	469

a cc-pVTZ basis set.

b The density functional is LRC-*ω*PBEh.

c 6-31G* basis set.

d The SOCC was computed between states |1〉 and |2〉.

Due to a weak ligand field, the five lowest states of Co(C(SiH_3_)_3_)_2_ are confined to a narrow energy range of only 5000 cm^−1^, while the sixth state is higher in energy at 15 000 cm^−1^. Both EOM-CCSD and CC2 yield the same ordering of the energies, with a deviation of CC2 from EOM-CCSD energies of at most 200 cm^−1^ for the low-lying states. However, this deviation increases to 700 cm^−1^ for the fifth and the sixth state. State |Ref_1_〉 is more stable than state |Ref_2_〉 by 500 cm^−1^ with EOM-CCSD and by 1300 cm^−1^ with CC2. When using EOM-CCSD-in-DFT, |Ref_1_〉 is again found to be more stable than |Ref_2_〉, by a similar magnitude. However, by employing EOM-CCSD-in-DFT, |Ref_1_〉 and |Ref_2_〉 become more stable than the two doubly-degenerate states |3〉/|4〉 and |3′〉/|4′〉, with the latter two states being almost degenerate. Additionally, states |5′〉 and |6′〉 are higher by 4000 cm^−1^ when computed with EOM-CCSD-in-DFT compared to EOM-CCSD and CC2. For the actual Co(C(SiMe_2_ONaph)_3_)_2_ molecular magnet, |Ref_2_〉 is more stable than |Ref_1_〉 by 2000 cm^−1^, contrary to what we observe for the model system Co(C(SiH_3_)_3_)_2_. Furthermore, the states |3〉/|4〉 and |3′〉/|4′〉 are flipped in energy with respect to Co(C(SiH_3_)_3_)_2_. Overall, the ground and first excited states are separated by 2500 cm^−1^ for the actual SMM, whereas the energy gap is much smaller for the model system, namely 800 cm^−1^ and 250 cm^−1^ with EOM-CCSD and CC2, respectively. Yet, in the case of the actual Co(ii)-SMM, where CC2 and EOM-CCSD calculations are not feasible, EOM-CCSD-in-DFT and NEVPT2 agree about the energetic ordering of all states (see Table S11, ESI†).

With EOM-CCSD, we compute an orbital angular momentum of 〈*L*_*z*_〉 ≈ 3*i* and a large SOCC of 1126 cm^−1^ between states |1〉 and |2〉. This is in agreement with the El-Sayed rule^103^ that predicts large SOCs between states with different orbital orientation, *i.e.*, from d_*xy*_ to d_*x*^2^−*y*^2^_, as well as from d_*xz*_ to d_*yz*_ (Fig. 9). Due to the SOC between states |1〉 and |2〉, the ground state splits into eight pairwise degenerate levels (Fig. 3). The corresponding energies are reported in Table S12 (ESI†). Using these levels, we computed an energy separation of 504 cm^−1^ between the ground and first-excited states, which is an estimate for the spin-reversal energy barrier.^15,94^ The agreement between EOM-CCSD and EOM-CCSD-in-DFT is very good with a deviation of 20 cm^−1^ for both the SOCC and spin–orbit splitting.

For Co(C(SiH_3_)_3_), truncation of the virtual orbital space only has a minor impact on the EOM-CCSD-in-DFT results. The energy differences are less than 100 cm^−1^ and the impact on the orbital angular momentum, SOCC, and spin-reversal barrier is negligible.

For Co(C(SiMe_2_ONaph)_3_)_2_, we computed an SOCC of 1059 cm^−1^, corresponding to an energy splitting between the ground and first excited state of 469 cm^−1^, which agrees very well with the value extracted from an FIR experiment^15^ (450 cm^−1^) and a value of 476 cm^−1^ from NEVPT2 calculations^15^ (see Table S12, ESI†). The differences in the computed SOCC and energy barrier between Co(C(SiH_3_)_3_)_2_ and Co(C(SiMe_2_ONaph)_3_)_2_ amount to only 50 cm^−1^. These results underscore that extending the electron correlation treatment from the Co^2+^ ion only in EOM-CCSD-in-DFT calculations to the entire complex with EOM-CCSD, or improving the structural model by incorporating naphthyl groups, does not alter the calculated properties associated with the SOC, as SOC is a local property.

**Fig. 10:**
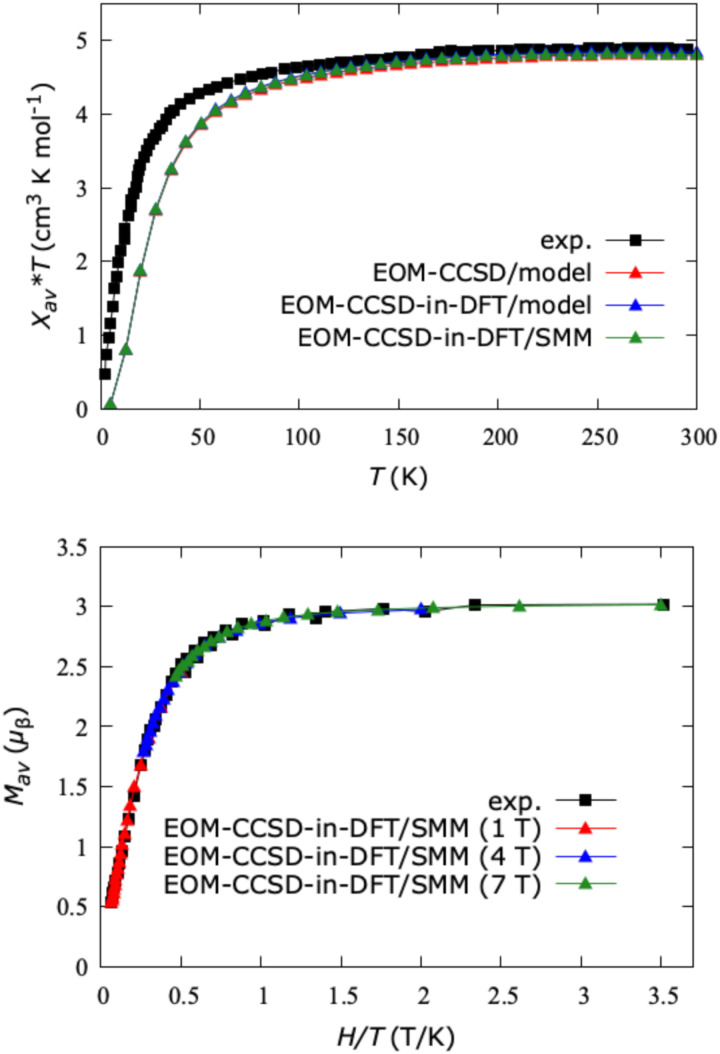
Top: Calculated susceptibility curves of the model system Co(C(SiH_3_)_3_)_2_ and actual SMM Co(C(SiMe_2_ONaph)_3_)_2_ between 5 and 300 K under an applied field of 7 T. Bottom: Calculated magnetizations for Co(C(SiMe_2_ONaph)_3_)_2_ at temperatures from 2 to 15 K under magnetic fields of 1, 4, and 7 T. “av” stands for isotropic powder averaging. Experimental data for Co(C(SiMe_2_ONaph)_3_)_2_ were taken from ref. 15. The density functional is LRC-*ω*PBEh.

Furthermore, using the magnetic sublevels of Co(C(SiH_3_)_3_)_2_ and Co(C(SiMe_2_ONaph)_3_)_2_, we computed temperature- and field-dependent susceptibilities (*χT vs. T*) and magnetizations (*M vs. H*/*T*). The results are illustrated in Fig. 10. The averaged data (“av”) reproduce the magnetic behavior of a powder sample and are determined through numerical integration, following the procedure presented in ref. 25. Our calculated curves exhibit excellent agreement with experiment^15^ and are nearly independent of the complex (Co(C(SiH_3_)_3_)_2_ or Co(C(SiMe_2_ONaph)_3_)_2_) and level of electron correlation (EOM-CCSD or EOM-CCSD-in-DFT). From the experiment at 300 K, the product of *χT* is 4.89 cm^3^ K mol^−1^, closely matching the computed *χ*_av_*T* of 4.80 cm^3^ K mol^−1^. These values are much higher than the spin-only value of 1.876 cm^3^ K mol^−1^ for an isotropic *S* = 3/2 ion, suggesting a large contribution of *L* to the magnetic behavior of the SMM. The agreement between theory and experiment is less good in the low-temperature regime; however, both curves show a significant deviation from the Curie law, that can again be interpreted as a consequence of the large orbital angular momentum. At 12 K and 7 T, we computed a saturation value of the magnetization of 3.01 *μ*_B_, also in agreement with the experimental value of 3.00 *μ*_B_. Furthermore, we did not observe a separation of the isofield magnetization curves (at 1, 4, and 7 T), which also confirms experimental observations. Overall, the calculated magnetization and susceptibility data are consistent with the magnetic behavior of a system with *S* = 3/2 and a sizeable contribution of the orbital angular momentum *L* = 3.

## IV. Conclusions

In this work, we assessed the performance of CC2 and EOM-CCSD-in-DFT for open-shell transition-metal complexes. Our test set included the [Fe(H_2_O)_6_]^2+^ and [Fe(H_2_O)_6_]^3+^ complexes, which were treated with the SF and EA variants of the CC methods. We also considered a Co(ii) single-molecule magnet, Co(C(SiMe_2_ONaph)_3_)_2_, and its simplified model system, Co(C(SiH_3_)_3_)_2_, for which we employed the EE variants of the methods. Our results show that CC2 outperforms EOM-CCSD-in-DFT for excitation energies. Also, we observe a substantial dependence of embedded EOM-CCSD energies on the density functional. Furthermore, truncating the virtual orbital space has only minimal impact on the energies of embedded EOM-SF-CCSD and EOM-EE-CCSD states, but a more pronounced effect on EOM-EA-CCSD energies. However, our results indicate that SOCCs, spin-reversal energy barriers, and macroscopic magnetic properties are insensitive to the choice of correlation scheme, EOM-CCSD or EOM-CCSD-in-DFT, and low-level DFT method, effectively reproducing experiment within spectroscopic accuracy. The adopted structural model, Co(C(SiMe_2_ONaph)_3_)_2_ or Co(C(SiH_3_)_3_)_2_, has no effect on the spin properties either. As a result, we conclude that using simpler structural models and employing an embedding potential for the interactions between the magnetic center and its environment have little effects on local properties such as the SOC.

The computed spin-reversal barrier for the Co(ii)-SMM ranges from 504 cm^−1^ for Co(C(SiH_3_)_3_)_2_ to 469 cm^−1^ for Co(C(SiMe_2_ONaph)_3_)_2_, representing a record value among transition-metal SMMs. This can be explained in terms of the El-Sayed rule^103^ and natural transition orbitals^25,67^ both of which predict large SOC in conjunction with a change of orbital orientation between the spin–orbit coupled states. Our calculated magnetizations and susceptibilities for Co(C(SiMe_2_ONaph)_3_)_2_ match experimental spectra well,^15^ consistently indicating a non-aufbau ground state with *J* = 9/2. These results do not depend on the electronic-structure method and are similar for the two cobalt complexes.

In sum, whereas embedded EOM-CCSD is less accurate than CC2 for excitation energies, our work illustrates its usefulness for spin-related properties. The embedding approach is versatile because the high-level fragment can be adapted to various properties of local character. Specifically, our results indicate that for molecular magnets whit spin density localized on the metal, ligand field effects—responsible for non-aufbau ground states and single-molecule magnetic behavior—can be adequately addressed by confining the high-level region solely to the metal ion. Additionally, different EOM-CCSD variants can be chosen for different electron configurations, which is particularly important for transition metals. Furthermore, with the integration of a periodic embedding potential, we envision that the resultant EOM-CCSD-in-DFT methods, in combination with the *ezMagnet* software, will find applications in the design of molecular magnetic materials, such as metal complexes adsorbed on a support or molecular self-assemblies.

## Conflicts of interest

There are no conflicts to declare.

## Supplementary Material

CP-026-D4CP01129E-s001

CP-026-D4CP01129E-s002

CP-026-D4CP01129E-s003

CP-026-D4CP01129E-s004

CP-026-D4CP01129E-s005
