# Supplementary material for: Coupled-cluster treatment of complex open-shell systems: the case of single-molecule magnets
Source: Phys Chem Chem Phys. 2024 May 27;26(24):17028–41. doi: 10.1039/d4cp01129e (PMC11186456; doi:10.1039/d4cp01129e)
Supplement: CP-026-D4CP01129E-s001 [file CP-026-D4CP01129E-s001.pdf]

Electronic Supplementary Material (ESI) for Physical Chemistry Chemical Physics.
This journal is © the Owner Societies 2024

Coupled-Cluster Treatment of Complex Open-Shell Systems: The Case of Single-Molecule Magnets

Maristella Alessio,[a,b,*] Garrette Pauley Paran,[a] Cansu Utku,[a] Andreas Grüneis[b] and Thomas-C. Jagau[a]

[a]Department of Chemistry, KU Leuven, Celestijnenlaan 200F, B-3001 Leuven, Belgium

[b]Institute for Theoretical Physics, TU Wien, Wiedner Hauptstraße 8-10/136, 1040 Vienna, Austria

* Corresponding author: maristella.alessio@kuleuven.be

Contents

1 Spin density S2

2 Wave function analysis of Fe-based systems S3

3 Additional CC2 results S6

4 Wave function analysis of Co-based systems S7

5 Effects of the low-level DFT method S10

6 Basis set effects S12

7 Comparison with additional benchmark data S14

1 Spin density

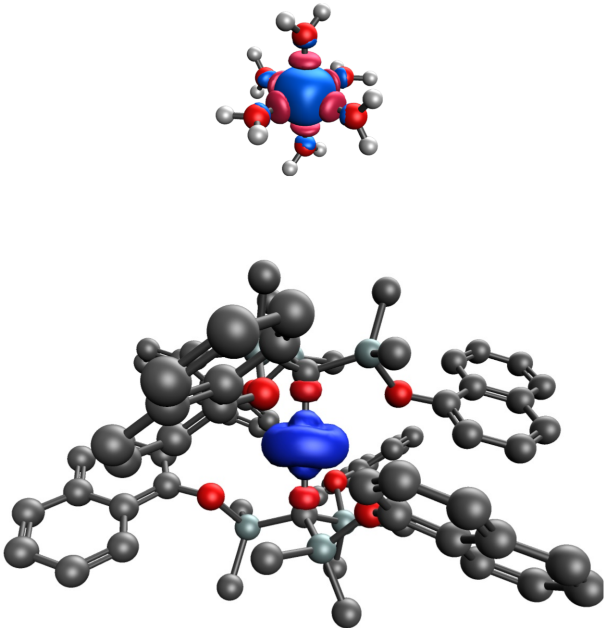

Figure S1: Spin density of $[Fe(H_2O)_6]^{3+}$ (top) and $Co(C(SiMe_2ONaph)_3)_2$ (bottom) (UHF/cc-pVTZ). For $Co(C(SiMe_2ONaph)_3)_2$, the hydrogen atoms have been omitted. An isovalue of 0.075 was used.

2 Wave function analysis of Fe-based systems

Table S1: EOM-EA-CCSD and EOM-EA-CCSD-in-LRC-ωPBEh/cc-pVTZ wave function properties of the five lowest eigenstates of $[Fe(H_2O)_6]^{2+}$. Energies are in cm^{-1}. EOM-EA-CCSD-in-LRC-ωPBEh/cc-pVTZ energies are obtained without truncation of the virtual space. Effective numbers of unpaired electrons ($n_{u,nl}$) are computed using Head-Gordon's formula.[1]

	EOM-EA-CCSD		
$\|1\rangle$	0	4.008	6.004
$\|1\rangle$	0	4.008	6.004
$\|2\rangle$	6	4.008	6.004
$\|3\rangle$	12	4.008	6.004
$\|4\rangle$	10905	4.929	6.004
$\|5\rangle$	10919	4.018	6.004

	EOM-EA-CCSD-in-LRC-ωPBEh		
State	E	$n_{u,nl}$	$\langle S^2\rangle$
$\|1\rangle$	0	4.006	6.074
$\|2\rangle$	7	4.006	6.074
$\|3\rangle$	13	4.006	6.074
$\|4\rangle$	11362	4.014	6.185
$\|5\rangle$	11378	4.014	6.185

Table S2: EOM-SF-CCSD and EOM-SF-CCSD-in-LRC-ωPBEh/cc-pVTZ wave function properties of the four lowest eigenstates of $[Fe(H_2O)_6]^{3+}$. Energies are in cm^{-1}. EOM-SF-CCSD-in-LRC-ωPBEh energies are obtained without truncation of the virtual space. Effective numbers of unpaired electrons ($n_{u,nl}$) are computed using Head-Gordon's formula.[1]

	EOM-SF-CCSD		
State	E	$n_{u,nl}$	$\langle S^2\rangle$
$\|1\rangle$	0	5.002	8.752
$\|2\rangle$	20314	3.017	3.771
$\|3\rangle$	20314	3.017	3.771
$\|4\rangle$	20314	3.017	3.771

	EOM-SF-CCSD-in-LRC-ωPBEh		
State	E	$n_{u,nl}$	$\langle S^2\rangle$
$\|1\rangle$	0	5.003	8.203
$\|2\rangle$	27218	3.486	3.821
$\|3\rangle$	27218	3.217	3.821
$\|4\rangle$	27218	3.483	3.821

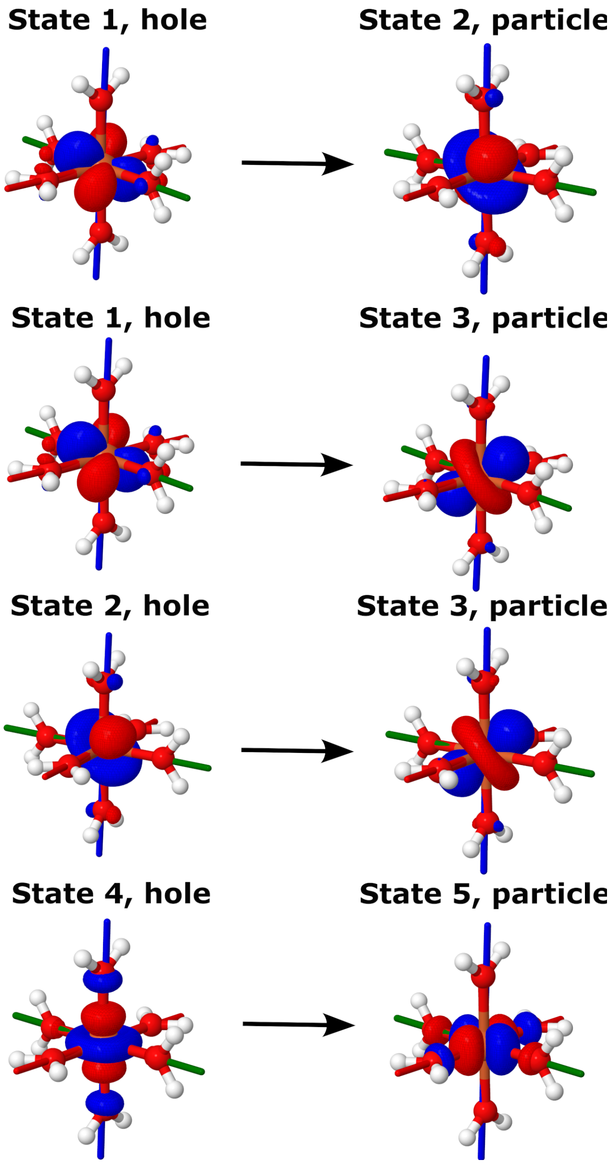

Figure S2: Hole and particle NTOs of the density matrix between states $|1\rangle$ and $|2\rangle$, $|1\rangle$ and $|3\rangle$, $|2\rangle$ and $|3\rangle$, and $|4\rangle$ and $|5\rangle$ (from top to bottom) of $[Fe(H_2O)_6]^{2+}$ computed with EOM-EA-CCSD-in-LRC-ωPBEh/cc-pVTZ. Red, green, and blue axes indicate x, y, and z axes. An isovalue of 0.05 was used.

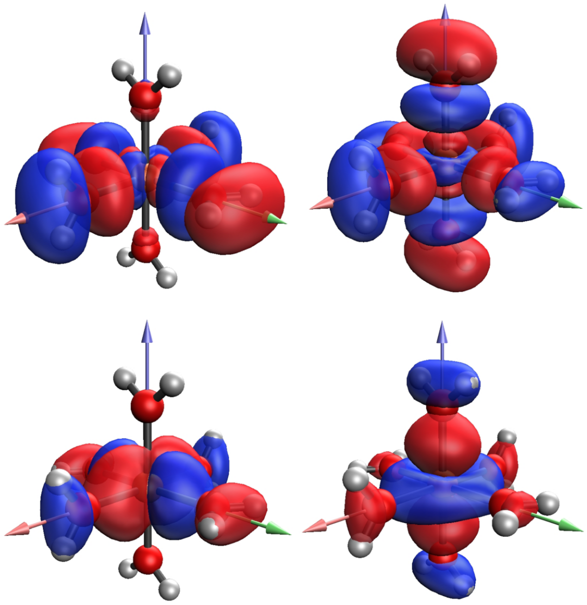

Figure S3: Anti-bonding (top) and bonding (bottom) HF/cc-pVTZ orbitals of $[Fe(H_2O)_6]^{3+}$, which result from the combination of the lone pair orbitals of water with the d_{z^2} and $d_{x^2-y^2}$ orbitals of Fe^{3+}. Red, green, and blue axes indicate x, y, and z axes. An isovalue of 0.05 was used.

3 Additional CC2 results

Table S3: Calculated CC2/cc-pVTZ excitation energy ΔE (eV) of $[Fe(H_2O)_6]^{2+}$ and $[Fe(H_2O)_6]^{3+}$, using Cholesky decomposition (CD) and the resolution-of-identity (RI) approximation. For $[Fe(H_2O)_6]^{2+}$, excitation energies are computed from states $|1\rangle$,$|2\rangle$,$|3\rangle$ to states $|4\rangle$,$|5\rangle$. For $[Fe(H_2O)_6]^{3+}$, excitation energies are computed from state $|1\rangle$ to states $|2\rangle$,$|3\rangle$,$|4\rangle$.

	$[Fe(H_2O)_6]^{2+}$			
	EA-CC2	EA-CD-CC2	EA-RI-CC2	EA-RI-SCS-CC2
ΔE	1.49	1.49	1.49	1.32
	$[Fe(H_2O)_6]^{3+}$			
	SF-CC2	SF-CD-CC2	SF-RI-CC2	
ΔE	2.49	2.48	2.49	

4 Wave function analysis of Co-based systems

Table S4: EOM-EE-CCSD and EOM-EE-CCSD-in-LRC-ωPBEh/cc-pVTZ wave function properties of the reference and target states of $Co(C(SiH_3)_3)_2$. Energies are in cm^{-1}. EOM-EE-CCSD-in-LRC-ωPBEh/cc-pVTZ energies are obtained without truncation of the virtual space. Effective numbers of unpaired electrons ($n_{u,nl}$) are computed using Head-Gordon's formula.[1]

	EOM-CCSD		
State	E	$n_{u,nl}$	$\langle S^2 \rangle$
$\|1\rangle$	0	3.332	3.756
$\|2\rangle$	11	3.333	3.756
$\|3\rangle$	803	3.010	3.758
$\|4\rangle$	803	3.010	3.758
$\|3'\rangle$	1855	3.275	3.757
$\|4'\rangle$	1855	3.275	3.757
$\|5'\rangle$	15609	3.111	3.763
$\|6'\rangle$	15609	3.111	3.763
$\|Ref_1\rangle$	3903	3.329	3.754
$\|Ref_2\rangle$	4427	3.336	3.754
	EOM-CCSD-in-LRC-ωPBEh		
State	E	$n_{u,nl}$	$\langle S^2 \rangle$
$\|1\rangle$	0	3.290	3.813
$\|2\rangle$	39	3.290	3.812
$\|3\rangle$	3287	3.008	3.924
$\|4\rangle$	3288	3.008	3.924
$\|3'\rangle$	3184	3.279	3.769
$\|4'\rangle$	3184	3.279	3.769
$\|5'\rangle$	19384	3.054	3.841
$\|6'\rangle$	19384	3.054	3.841
$\|Ref_1\rangle$	1089	3.012	3.753
$\|Ref_2\rangle$	4964	3.017	3.751

Table S5: Occupations of frontiers natural orbitals n_β and n_α of the reference ($|\mathrm{Ref}_1\rangle$ and $|\mathrm{Ref}_2\rangle$) and doubly-degenerate ground state ($|1\rangle$ and $|2\rangle$) of $Co(C(SiH_3)_3)_2$ (EOM-EE-CCSD-in-LRC-ωPBEh/cc-pVTZ).

$|1\rangle$	d_{xy}	$d_{x^2-y^2}$	d_{xz}	d_{yz}	d_{z^2}
n_α	1.00	1.00	1.00	1.00	1.00
n_β	0.50	0.50	0.49	0.49	
$|2\rangle$	d_{xy}	$d_{x^2-y^2}$	d_{xz}	d_{yz}	d_{z^2}
n_α	1.00	1.00	1.00	1.00	1.00
n_β	0.50	0.50	0.49	0.49	
$|\mathrm{Ref}_1\rangle$	d_{xy}	$d_{x^2-y^2}$	d_{xz}	d_{yz}	d_{z^2}
n_α	0.99	0.99	0.99	0.99	0.99
n_β	0.97	0.97			
$|\mathrm{Ref}_2\rangle$	d_{xy}	$d_{x^2-y^2}$	d_{xz}	d_{yz}	d_{z^2}
n_α	0.99	0.99	0.99	0.99	0.99
n_β			0.96	0.96	

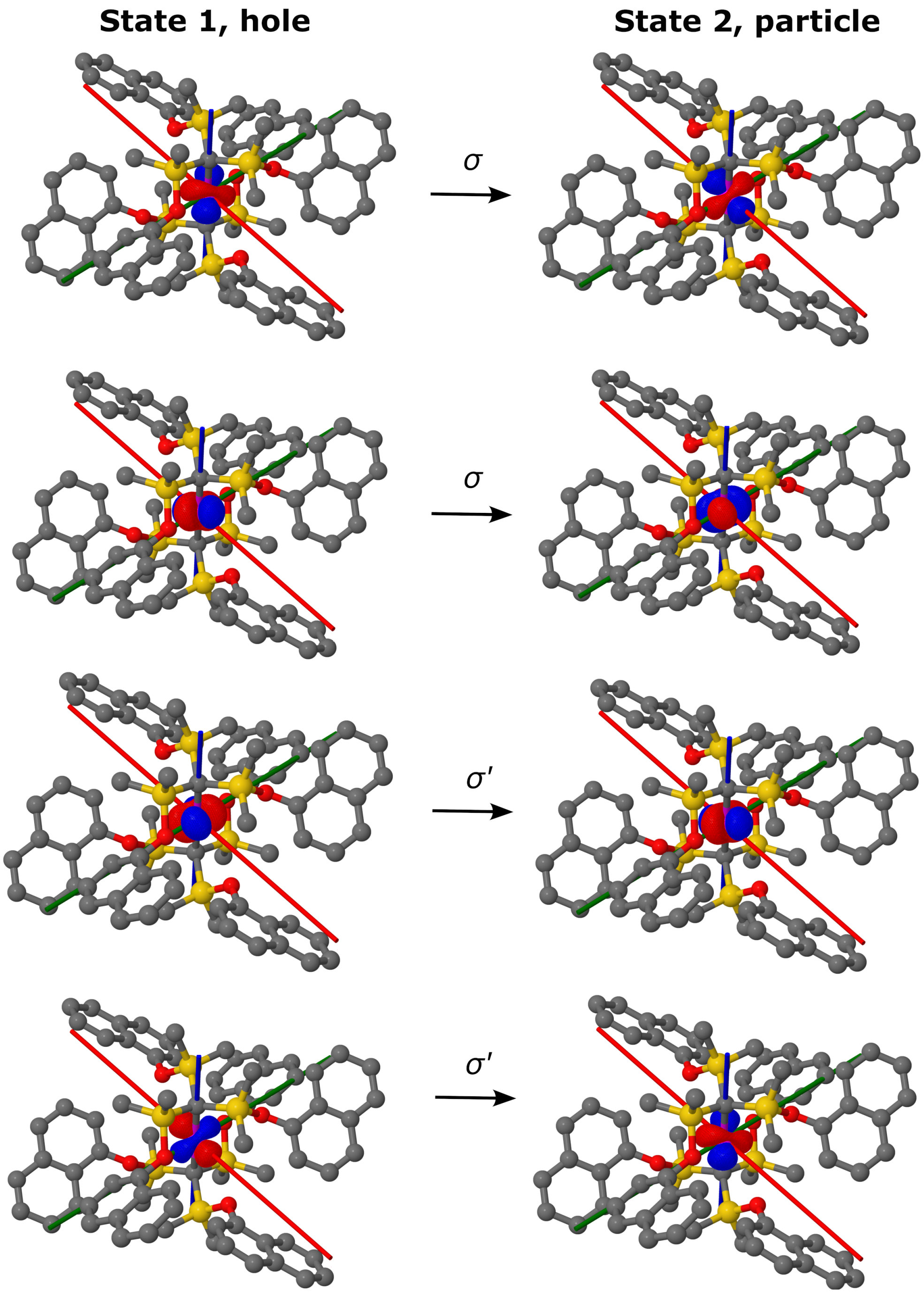

Figure S4: Hole and particle NTOs for SOC between states $|1\rangle$ and $|2\rangle$ of $Co(C(SiMe_2ONaph)_3)_2$ (EOM-EE-CCSD-in-LRC-ωPBEh/6-31G*). Singular values are $\sigma = 0.49$ and $\sigma' = 0.42$. Red, green, and blue axes indicate x, y, and z axes. An isovalue of 0.05 was used.

5 Effects of the low-level DFT method

Table S6: Excitation energies ΔE (eV) and spin-orbit coupling constants SOCCs (cm^{-1}) of $[Fe(H_2O)_6]^{2+}$ obtained using EOM-EA-CCSD and EOM-EA-CCSD-in-DFT with cc-pVTZ basis set. Excitation energies are computed from states $|1\rangle$,$|2\rangle$,$|3\rangle$ to states $|4\rangle$,$|5\rangle$. SOCCs are computed between states $|1\rangle$ and $|2\rangle$, $|1\rangle$ and $|3\rangle$, and $|2\rangle$ and $|3\rangle$. EOM-EA-CCSD-in-DFT energies are obtained without truncation of the virtual space.

	EOM-EA-CCSD
ΔE	1.35
SOCC	282

	EOM-EA-CCSD-in-DFT					
	PBE0	LRC-ωPBEh	B3LYP	B5050LYP	CAM-B3LYP	ωB97x-D
ΔE	1.29	1.41	0.94	0.96	0.97	1.83
SOCC	235	242	237	265	250	218

Table S7: Excitation energies ΔE (eV) and spin-orbit coupling constants SOCCs (cm^{-1}) of $[Fe(H_2O)_6]^{3+}$ obtained using EOM-SF-CCSD, EOM-SF-CCSD-in-DFT, and SF-TDDFT with cc-pVTZ basis set. Excitation energies are computed from state $|1\rangle$ to states $|2\rangle$,$|3\rangle$,$|4\rangle$. SOCCs are computed between state $|1\rangle$ and the triply-degenerate excited state (i.e., states $|2\rangle$, $|3\rangle$, $|4\rangle$). EOM-SF-CCSD-in-DFT energies are obtained without truncation of the virtual space.

	EOM-SF-CCSD
ΔE	2.52
SOCC	839

	EOM-SF-CCSD-in-DFT					
	PBE0	LRC-ωPBEh	B3LYP	B5050LYP	CAM-B3LYP	ωB97x-D
ΔE	3.37	3.37	3.37	3.63	3.45	3.29
SOCC	889	889	878	893	882	895

	SF-DFT					
	PBE0	LRC-ωPBEh	B3LYP	B5050LYP	CAM-B3LYP	ωB97x-D
ΔE	2.02	2.01	1.49	1.91	1.51	1.63
SOCC	662	659	665	757	686	685

Table S8: Energies of electronic states (cm^{-1}) and spin-orbit coupling constants SOCCs (cm^{-1}) of $Co(C(SiH_3)_3)_2$ using EOM-EE-CCSD, CD-EE-CC2, and EOM-EE-CCSD-in-DFT with cc-pVTZ. DFT is LRC-ωPBEh and CAM-B3LYP. EOM-EE-CCSD-in-DFT energies are obtained without truncation of the virtual space. SOCC is between states $|1\rangle$ and $|2\rangle$.

	$Co(C(SiH_3)_3)_2$			
	EOM-CCSD	CD-CC2	EOM-CCSD-in-LRC-ωPBEh	EOM-CCSD-in-CAM-B3LYP
$\|1\rangle$	0	0	0	0
$\|2\rangle$	11	4	39	97
$\|3\rangle$	803	258	3287	3193
$\|4\rangle$	803	261	3288	3193
$\|3'\rangle$	1855	1645	3184	3185
$\|4'\rangle$	1855	1657	3184	3185
$\|Ref_1\rangle$	3903	3690	1128	650
$\|Ref_2\rangle$	4427	4964	2412	2597
$\|5'\rangle$	15609	14911	19384	19812
$\|6'\rangle$	15609	14914	19384	19812
$\langle 1\|L_z\|2\rangle$	$2.98i$		$3.01i$	$3.16i$
SOCC	1126		1107	1162

6 Basis set effects

Table S9: Excitation energies ΔE (eV) and spin-orbit coupling constants SOCCs (cm^{-1}) of $[Fe(H_2O)_6]^{2+}$ and $[Fe(H_2O)_6]^{3+}$ obtained using EOM-CCSD, EOM-CCSD-in-LRC-ωPBEh, and CC2 with cc-pVDZ and cc-pVTZ basis sets. SOCC of $[Fe(H_2O)_6]^{2+}$ is between states $|1\rangle$ and $|2\rangle$, $|1\rangle$ and $|3\rangle$, and $|2\rangle$ and $|3\rangle$. SOCC of $[Fe(H_2O)_6]^{3+}$ is between state $|1\rangle$ and the triply-degenerate excited state (i.e., states $|2\rangle$, $|3\rangle$, $|4\rangle$). EOM-CCSD-in-DFT energies are obtained with and without (in parenthesis) truncation of the virtual space.

	$[Fe(H_2O)_6]^{2+}$					
	EOM-EA-CCSD		EA-CC2		EOM-EA-CCSD-in-LRC-ωPBEh	
	cc-pVDZ	cc-pVTZ	cc-pVDZ	cc-pVTZ	cc-pVDZ	cc-pVTZ
ΔE^a	1.33	1.35	1.48	1.49	1.41 (1.51)	1.41 (1.57)
SOCC	278	282			233 (242)	242 (252)

	$[Fe(H_2O)_6]^{3+}$					
	EOM-SF-CCSD		SF-CC2		EOM-SF-CCSD-in-LRC-ωPBEh	
	cc-pVDZ	cc-pVTZ	cc-pVDZ	cc-pVTZ	cc-pVDZ	cc-pVTZ
ΔE^b	2.56	2.52	2.53	2.49	3.45 (3.45)	3.37 (3.38)
SOCC	828	839			877 (877)	890 (890)

^{a} $|1\rangle,|2\rangle,|3\rangle \rightarrow |4\rangle,|5\rangle$. ^{b} $|1\rangle \rightarrow |2\rangle,|3\rangle,|4\rangle$.

Table S10: Energies of electronic states (cm^{-1}) of $Co(C(SiH_3)_3)_2$ and $Co(C(SiMe_2ONaph)_3)_2$ computed using EOM-EE-CCSD, CD-EE-CC2, and EOM-EE-CCSD-in-DFT with 6-31G*, def2-SV(P), and cc-pVTZ basis sets. The density functional is LRC-ωPBEh. For $Co(C(SiMe_2ONaph)_3)_2$, EOM-EE-CCSD-in-DFT results are available for the truncated virtual space only.

	$Co(C(SiH_3)_3)_2$					
	EOM-CCSD		CD-CC2		EOM-CCSD-in-LRC-ωPBEh	
	6-31G*	cc-pVTZ	6-31G*	cc-pVTZ	6-31G*	cc-pVTZ
$\|Ref_2\rangle$	4129	4427	5473	4964	2847	2412
$\|1\rangle$	0	0	0	0	0	0
$\|2\rangle$	1	10	0	1	0	22
$\|3'\rangle$	1102	1855	866	1645	2531	3184
$\|4'\rangle$	1102	1855	866	1657	2532	3184
$\|5'\rangle$	14931	15609	14625	14911	17565	19384
$\|6'\rangle$	14930	15609	14625	14914	17564	19384

	$Co(C(SiMe_2ONaph)_3)_2$	
	EOM-CCSD-in-LRC-ωPBEh	
	6-31G*	cc-pVTZ
$\|Ref_2\rangle$	2492	1778
$\|1\rangle$	0	0
$\|2\rangle$	45	15
$\|3'\rangle$	2721	3110
$\|4'\rangle$	2722	3111
$\|5'\rangle$	19180	20252
$\|6'\rangle$	19184	20255

7 Comparison with additional benchmark data

Table S11: Energies of electronic states (cm^{-1}), spin-orbit coupling constants SOCCs (cm^{-1}), and spin-inversion energy barriers U (cm^{-1}) of $Co(C(SiH_3)_3)_2$ and $Co(C(SiMe_2ONaph)_3)_2$ computed using EOM-EE-CCSD, CD-EE-CC2, and EOM-EE-CCSD-in-DFT with cc-pVTZ. The density functional is LRC-ωPBEh. EOM-EE-CCSD-in-DFT energies are obtained with truncation of the virtual space. SOCC is for the doubly-degenerate ground state (i.e., $|1\rangle$ and $|2\rangle$). NEVPT2 energies are taken from Ref. [2].

	$Co(C(SiH_3)_3)_2$			$Co(C(SiMe_2ONaph)_3)_2$	
	EOM-CCSD^a	CD-CC2^a	EOM-CCSD-in-DFT^a	EOM-CCSD-in-DFT^b	NEVPT2
$\|1\rangle$	0	0	0	0	0
$\|2\rangle$	11	4	46	20	53
$\|3\rangle$	803	258	3225	3556	2768
$\|4\rangle$	803	261	3226	3556	2768
$\|3'\rangle$	1855	1645	3187	2721	2014
$\|4'\rangle$	1855	1657	3187	2722	2014
$\|Ref_1\rangle$	3903	3690	1009	4489	13537
$\|Ref_2\rangle$	4427	4964	2400	2492	1500
$\|5'\rangle$	15609	14911	19406	19180	18865
$\|6'\rangle$	15609	14914	19407	19184	18865
$\langle 1\|L_z\|2\rangle$	$2.98i$		$3.02i$	$2.99i$	
SOCC	1126		1114	1050	
U	504		497	469	476

^{a} cc-pVTZ basis set. ^{b} 6-31G* basis set.

Table S12: Spin–orbit splitting of the doubly-degenerate ground state ($|1\rangle$ and $|2\rangle$) of $Co(C(SiH_3)_3)_2$ and $Co(C(SiMe_2ONaph)_3)_2$. Energies of the sublevels are in cm^{-1}. EOM-EE-CCSD-in-LRC-ωPBEh/cc-pVTZ energies are obtained with truncation of the virtual space. NEVPT2 energies are taken from Ref. [2].

	$Co(C(SiH_3)_3)_2$		$Co(C(SiMe_2ONaph)_3)_2$	
	EOM-CCSD^a	EOM-CCSD-in-DFT^a	EOM-CCSD-in-DFT^b	NEVPT2
$M_J = \pm 9/2$	0	0	0	0
$M_J = \pm 7/2$	504	497	469	476
$M_J = \pm 5/2$	1007	997	941	969
$M_J = \pm 3/2$	1511	1495	1410	1469

^{a} cc-pVTZ basis set. ^{b} 6-31G* basis set.

References

[1] M. Head-Gordon. Characterizing unpaired electrons from the one-particle density matrix. *Chem. Phys. Lett.*, 372:508–511, 2003.

[2] P. C. Bunting, M. Atanasov, E. Damgaard-Møller, M. Perfetti, I. Crassee, M. Orlita, J. Overgaard, J. van Slageren, F. Neese, and J. R. Long. A linear cobalt(II) complex with maximal orbital angular momentum from a non-Aufbau ground state. *Science*, 362(6421):2146–2149, 2018.

Relevant Cartesian coordinates

```
$comment
[Fe(H2O)6]^3+, wB97X-D/cc-pVDZ (C1)
$end

$molecule
Fe      0.0002361677     0.0000476251     0.0009397389
O      -0.0721189257     0.0489498845    -2.0388231562
O       0.0737598235    -0.0476929346     2.0414469592
O       0.0549164873     2.0401098023     0.0486304137
O      -2.0389792585     0.0524780849     0.0756714441
O      -0.0547818719    -2.0400529569    -0.0453185578
O       2.0396533543    -0.0528612399    -0.0711812427
H      -2.6462604464    -0.7138298032     0.0788015817
H      -2.6044271001     0.8490518214     0.1192386544
H       2.6050615147    -0.8495994235    -0.1120905241
H       2.6470815104     0.7133260007    -0.0744189591
H       0.8765686589    -0.0851726048     2.5983947722
H      -0.6854593633    -0.0421063530     2.6574324088
H       0.6876662666     0.0432232602    -2.6541932271
H      -0.8746082195     0.0843400673    -2.5964555133
H       0.0983430455     2.6073634271     0.8439219965
H       0.0421180234     2.6460438162    -0.7186352159
H      -0.0445481784    -2.6456058663     0.7222890300
H      -0.0952794885    -2.6077686075    -0.8404376031
$end

$comment
[Fe(H2O)6]^3+, wB97X-D/cc-pVDZ (D2h)
$end

$molecule
Fe      0.0000000000     0.0000000000     0.0000000000
O       0.0000000000     0.0000000000     2.0417922000
O       0.0000000000    -2.0417922000     0.0000000000
O       2.0417922000     0.0000000000     0.0000000000
O       0.0000000000     2.0417922000     0.0000000000
O       0.0000000000     0.0000000000    -2.0417922000
O      -2.0417922000     0.0000000000     0.0000000000
H       0.0000000000     0.7819742000     2.6288140000
H       0.0000000000    -0.7819742000     2.6288140000
H       2.6288140000     0.0000000000     0.7819742000
H       2.6288140000     0.0000000000    -0.7819742000
H       0.7819742000     2.6288140000     0.0000000000
H      -0.7819742000     2.6288140000     0.0000000000
```

```
H       0.0000000000    -0.7819742000    -2.6288140000
H       0.0000000000     0.7819742000    -2.6288140000
H      -2.6288140000     0.0000000000    -0.7819742000
H      -2.6288140000     0.0000000000     0.7819742000
H      -0.7819742000    -2.6288140000     0.0000000000
H       0.7819742000    -2.6288140000     0.0000000000
$end

$comment
Co(C(SiH3)3)2, wB97X-D/cc-pVDZ (C2h)
$end

$molecule
Co     -0.0000000000     0.0000000000    -0.0000000000
C      -1.9836800000     0.0000028832    -0.0000000000
C       1.9836800000    -0.0000028832    -0.0000000000
Si      2.5504386917    -0.9001237071    -1.5590600000
Si      2.5504386917    -0.9001237070     1.5590600000
Si      2.5504426166     1.8002462930    -0.0000000000
Si     -2.5504426166    -1.8002462930     0.0000000000
Si     -2.5504386917     0.9001237070    -1.5590600000
Si     -2.5504386917     0.9001237071     1.5590600000
H       2.0227666497    -2.3050229401    -1.5678500000
H       4.0443285993    -0.9636558784    -1.6691000000
H       2.0227697016    -0.2052929401    -2.7801300000
H       2.0227666497    -2.3050229400     1.5678500000
H       2.0227697016    -0.2052929400     2.7801300000
H       4.0443285993    -0.9636558783     1.6691000000
H       2.0227736487     2.5103070600     1.2122800000
H       2.0227736487     2.5103070599    -1.2122800000
H       4.0443328013     1.9273041216    -0.0000000000
H      -2.0227736487    -2.5103070600    -1.2122800000
H      -4.0443328013    -1.9273041216     0.0000000000
H      -2.0227736487    -2.5103070599     1.2122800000
H      -2.0227697016     0.2052929400    -2.7801300000
H      -2.0227666497     2.3050229400    -1.5678500000
H      -4.0443285993     0.9636558783    -1.6691000000
H      -2.0227697016     0.2052929401     2.7801300000
H      -4.0443285993     0.9636558784     1.6691000000
H      -2.0227666497     2.3050229401     1.56785000001
$end
```
