# Supplementary figures and images for: Coupled-cluster treatment of complex open-shell systems: the case of single-molecule magnets
Source: Phys Chem Chem Phys. 2024 May 27;26(24):17028–41. doi: 10.1039/d4cp01129e (PMC11186456; doi:10.1039/d4cp01129e)

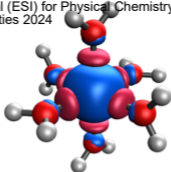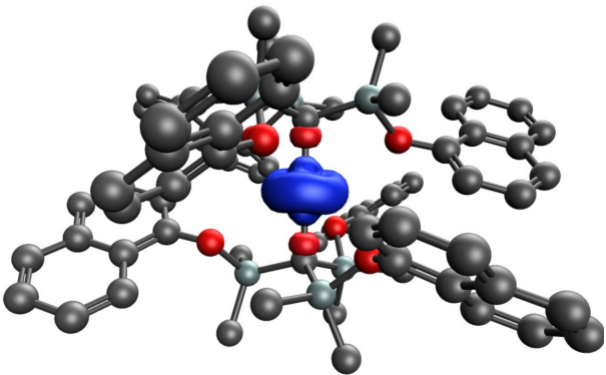

Supplement: CP-026-D4CP01129E-s002 [file CP-026-D4CP01129E-s002.pdf]

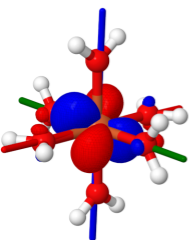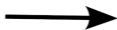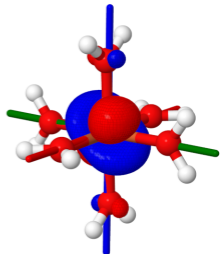

State 1, hole

State 3, particle

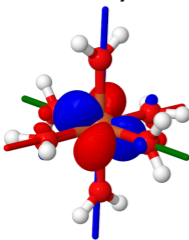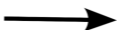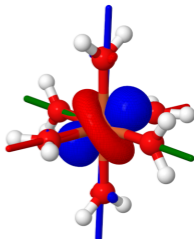

State 2, hole

State 3, particle

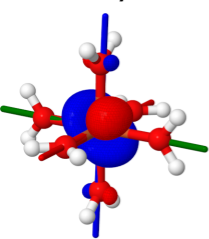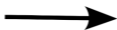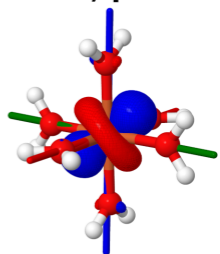

State 4, hole

State 5, particle

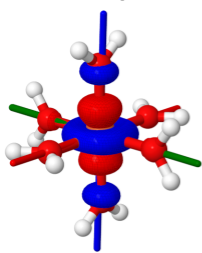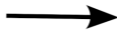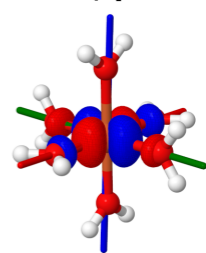

Supplement: CP-026-D4CP01129E-s003 [file CP-026-D4CP01129E-s003.pdf]

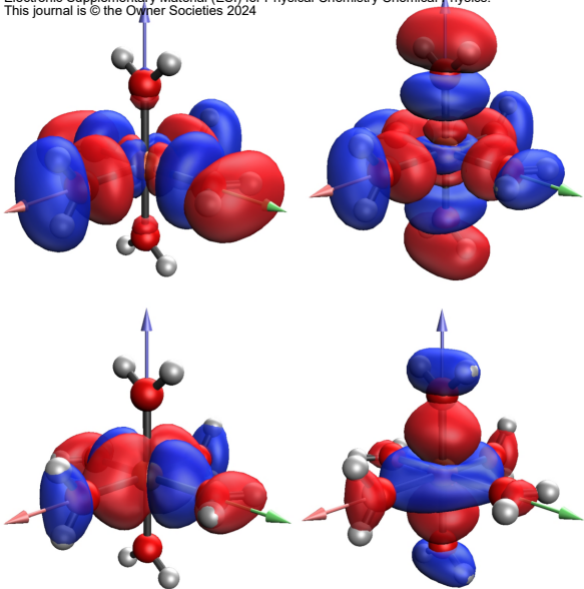

Supplement: CP-026-D4CP01129E-s004 [file CP-026-D4CP01129E-s004.pdf]

**State 1, hole**

**State 2, particle**

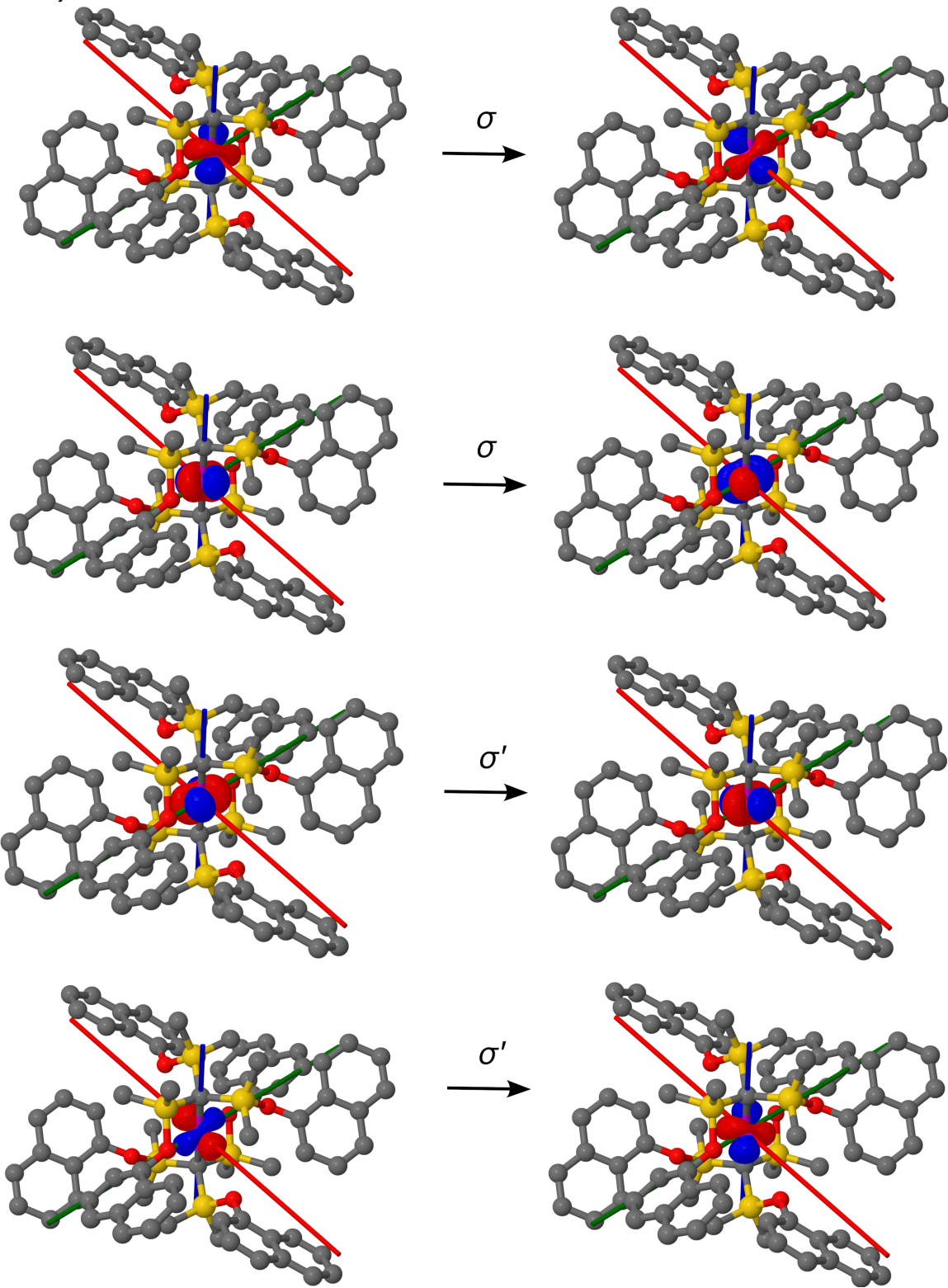

Supplement: CP-026-D4CP01129E-s005 [file CP-026-D4CP01129E-s005.pdf]
